# Comparative Analysis of Human Tissue Interactomes Reveals Factors Leading to Tissue-Specific Manifestation of Hereditary Diseases

**DOI:** 10.1371/journal.pcbi.1003632

**Published:** 2014-06-12

**Authors:** Ruth Barshir, Omer Shwartz, Ilan Y. Smoly, Esti Yeger-Lotem

**Affiliations:** 1 Department of Clinical Biochemistry and Pharmacology, Ben-Gurion University of the Negev, Beer-Sheva, Israel; 2 Department of Computer Science, Ben-Gurion University of the Negev, Beer-Sheva, Israel; 3 National Institute for Biotechnology in the Negev, Ben-Gurion University of the Negev, Beer-Sheva, Israel; University of Washington, United States of America

## Abstract

An open question in human genetics is what underlies the tissue-specific manifestation of hereditary diseases, which are caused by genomic aberrations that are present in cells across the human body. Here we analyzed this phenomenon for over 300 hereditary diseases by using comparative network analysis. We created an extensive resource of protein expression and interactions in 16 main human tissues, by integrating recent data of gene and protein expression across tissues with data of protein-protein interactions (PPIs). The resulting tissue interaction networks (interactomes) shared a large fraction of their proteins and PPIs, and only a small fraction of them were tissue-specific. Applying this resource to hereditary diseases, we first show that most of the disease-causing genes are widely expressed across tissues, yet, enigmatically, cause disease phenotypes in few tissues only. Upon testing for factors that could lead to tissue-specific vulnerability, we find that disease-causing genes tend to have elevated transcript levels and increased number of tissue-specific PPIs in their disease tissues compared to unaffected tissues. We demonstrate through several examples that these tissue-specific PPIs can highlight disease mechanisms, and thus, owing to their small number, provide a powerful filter for interrogating disease etiologies. As two thirds of the hereditary diseases are associated with these factors, comparative tissue analysis offers a meaningful and efficient framework for enhancing the understanding of the molecular basis of hereditary diseases.

## Author Summary

An open question in human genetics is what underlies the tissue-specific manifestation of hereditary diseases, which are caused by genomic aberrations that are present in cells across the entire human body. In order to answer this question, we created an extensive resource of protein expression and interactions across 16 main human tissues. Using this resource, we first show that the genes underlying hundreds of hereditary diseases are widely expressed across tissues, yet, enigmatically, cause disease phenotypes in few tissues only. We then identify two distinct, statistically-significant factors that could lead to tissue-specific vulnerability in the face of this broad expression: (i) many disease-causing genes have elevated expression levels in their disease tissues, and (ii) disease-causing genes have a significantly higher tendency for tissue-specific interactions in their disease tissues. As we show for several disease-causing genes, these tissue-specific interactions highlight disease mechanisms and provide an efficient filter for interrogating the molecular basis of diseases. Together the two factors we identified are relevant for as many as two thirds of the tissue-specific hereditary diseases. Our comparative tissue analysis therefore provides a meaningful and efficient framework for enhancing the understanding of the molecular basis of hereditary diseases.

## Introduction

Hereditary diseases arise due to germline aberrations that are present across the human body. Enormous progress has been made over the years in mapping the genetic causes for a large variety of hereditary diseases. To date, causal germline aberrations for over 1,500 hereditary diseases can be retrieved from the OMIM database [1]. Additional disease-related factors have been mapped by genome-wide association studies, mRNA profiling and epigenetic marking. Yet, despite this wealth of data, the molecular basis of many hereditary diseases remains elusive. It is thus apparent that novel strategies are required to enhance our understanding of the molecular basis of these diseases.

While the genetic aberrations that cause hereditary diseases are global, the diseases are often manifested in specific organs or tissues (for simplicity we will use ‘tissue’ to denote ‘organ’ as well). The mechanism for this selectivity and vulnerability in certain tissues is unknown. Common explanations refer to a unique function of the disease tissue, as in the case of muscle and liver glycogen storage disease, or a unique feature of the disease tissue, such as long-lived neurons and age-related protein misfolding diseases [2]. It was also shown that expression levels of genes underlying genetic diseases tend to be elevated in their disease tissues [3], [4]. Yet a rigorous analysis of the tissue-specific manifestation of hereditary diseases and their respective disease genes has rarely been performed.

An important determinant of tissue features is the repertoire of proteins that are expressed in the tissue and their physical interactions, whose union defines the tissue interactome. Tissue interactomes were utilized to assess tissue-specific functions of proteins and interactions (e.g., [4]–[8]) and to illuminate general properties of disease genes (e.g., [3], [7]). Tissue interactomes were typically constructed by superimposing tissue expression data on a static protein-protein interaction (PPI) network composed of interactions that were frequently identified through *in-vitro* assays, without any tissue context. These PPIs were included in the tissue interactome if the interacting partners were found to be expressed in that tissue, otherwise the PPIs were down-weighted or excluded (e.g., [4]–[11]). Due to the scarcity of protein expression data across tissues, most studies exploited data of gene expression across tissues. The dataset of Su et al. [12], which used DNA microarrays to profile transcript levels across 77 tissues, has been a prominent quantitative resource in many of these studies (e.g., [3], [9], [11]).

Recently, our views of gene and protein expression across tissues were considerably amplified owing to the application of additional technologies to expression mapping. First, application of next-generation RNA sequencing (RNA-seq) revealed that many more transcripts were expressed per tissue than previously acknowledged [13], thereby affecting conclusions drawn from previous data [7]. Second, large-scale measurements of protein abundance have become available, providing direct evidence for the presence of a protein in a tissue. In particular, the Human Protein Atlas (HPA) initiative offers qualitative immunohistochemical measurements of protein abundance for thousands of proteins across tens of tissues [14].

**Figure 1:**
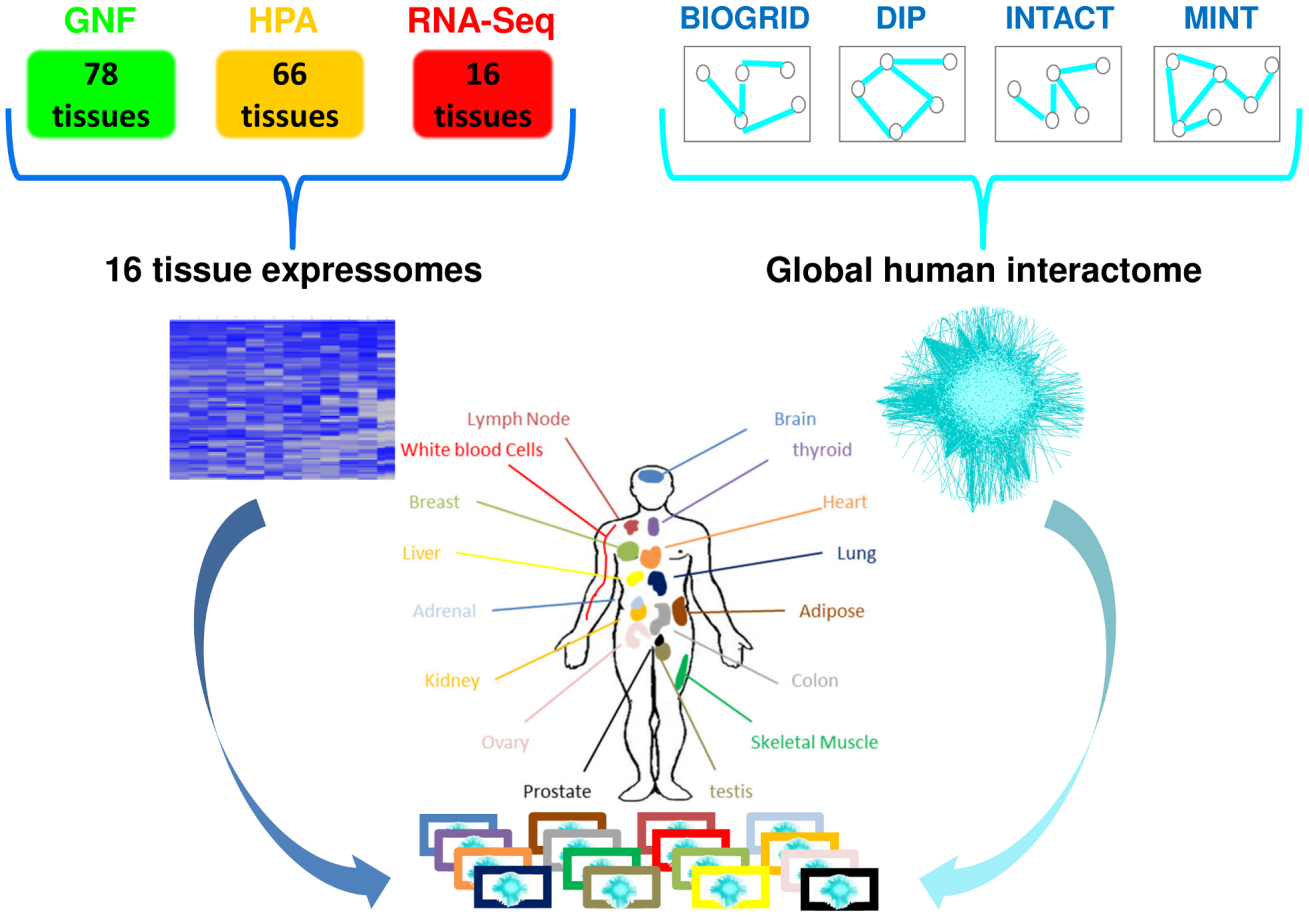
The construction of 16 human tissue interactomes by integrating data of tissue expression with data of PPIs. Data of expression per tissue according to DNA microarray (GNF, [12]), protein abundance (HPA, [14]), and RNA-sequencing (RNA-seq, [15]) were consolidated into 16 main tissues. In parallel, experimentally detected PPIs were united from BIOGRID [16], DIP [17], IntAct [18] and MINT [19] to form a global human interactome. Tissue interactomes were then constructed by filtering the global interactome per tissue to contain only PPIs in which both pair-mates were found to be expressed within the tissue.

Here we took advantage of the exciting new wealth of information regarding gene and protein expression across tissues and harnessed them to illuminate the tissue-specificity of hereditary diseases. First, we combined these data and integrated them with known PPIs to create the interactomes of 16 main human tissues (Figure 1). We observed considerable similarities between tissue interactomes in terms of expressed proteins and interactions, including significant correlations between transcript levels and the numbers of interacting partners per gene in each tissue. Focusing on genes causing hereditary diseases, we show that, in contrast to the tissue-specific manifestation of their respective diseases, the genes are often expressed in many tissues. However, they tend to have elevated transcript levels or tissue-specific PPIs preferentially in their disease tissues. We demonstrate that knowledge of the tissue-specific PPIs of genes causing hereditary diseases can be used to highlight disease-related mechanisms. Therefore, comparison between tissue interactomes can serve as an efficient strategy for illuminating the molecular basis of these diseases.

## Results

### Tissue profiling data reveals a bi-modal distribution of expressed genes across tissues

We obtained extensive data of genes and proteins expression across tissues by integrating three major datasets: the dataset of the Genomics Institute of the Novartis Research Foundation (GNF) measured by using DNA microarrays [12], the Human Protein Atlas (HPA) dataset described above [14], and the Illumina Body Map 2.0 dataset measured by using RNA-seq [15]. From each dataset we extracted the set of proteins or protein-coding genes expressed per tissue, denoted tissue expressome, by using stringent thresholds (see Methods). Since the available PPI data were oblivious to alternatively-spliced isoforms we associated each protein-coding gene with a single protein product (see Methods). For simplicity we henceforth refer to protein-coding genes and proteins interchangeably.

**Figure 2:**
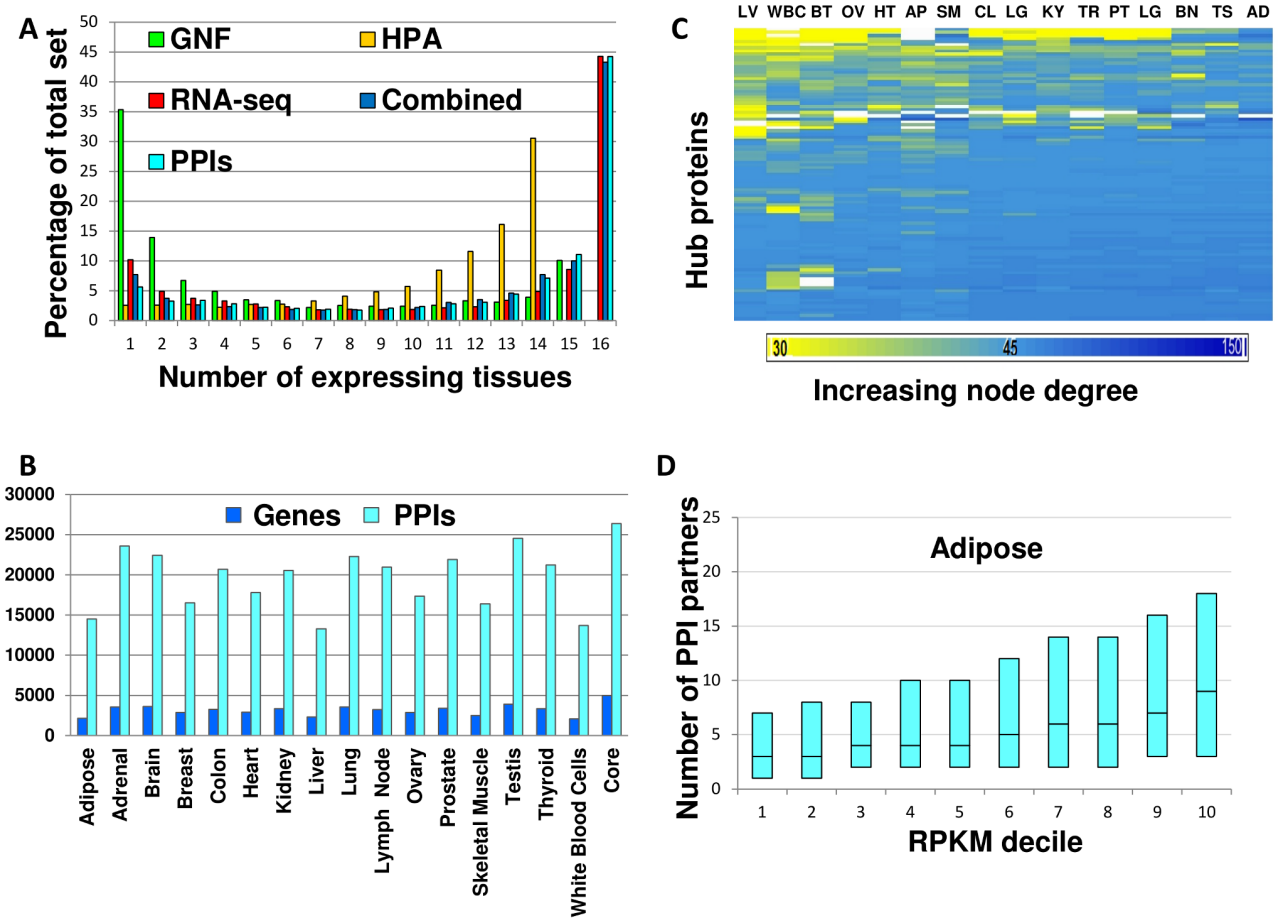
Common features of tissue interactomes. A. The distribution of proteins and PPIs by the number of tissues in which they are expressed is bi-modal, with most genes being globally expressed or tissue-specific. The distribution is shown per dataset and when combined. PPIs show a corresponding bi-modal distribution (the numbers of PPIs across 1–16 tissues appear in Table S11). B. A comparative view of the numbers of expressed proteins and PPIs across tissues. The core sub-network that is shared by all tissues (the right-most bar) is larger than the interactome of each tissue that remains after excluding the core. The numbers of genes and PPIs in the interactome of each tissue appear in Table S12. C. Most tissue hubs are widely expressed and retain their large PPI degree when expressed. The PPI degrees of the 451 tissue hubs (rows) in the 16 tissue interactomes (columns) are presented using a heat map, where each entry marks the PPI degree of the corresponding hub in that tissue. Entries are colored by the PPI degree from yellow (≤30 PPIs) to dark blue (≥150 PPIs); a white entry implies that the hub is not expressed in that tissue. Tissue acronyms: LV = Liver, WBC = White Blood Cells, BT = Breast, OV = Ovary, HT = Heart, AP = Adipose, SM = Skeletal Muscle, CL = Colon, LG = Lung, KY = Kidny, TR = Thyroid, PT = Prostate, LG = Lung, BN = Brain, TS = Testis, AD = Adrenal. D. A strong correlation between RPKM levels and PPI degree is observed in adipose tissue (Spearman r = 0.98, p = 4.7*10^−7^). The box-plot diagram shows the quartiles (25%, 50% and 75%) of the sorted PPI degree values in each RPKM bin. Similar correlations were observed in all 16 tissues (Figure S3).

Dataset comparison of the genes expressed in each tissue revealed that RNA-seq identified the largest number of genes per tissue, with a median increase of 1.5-fold relative to HPA and of 3.8-fold relative to GNF (Table 1). Still, RNA-seq did not fully contain data from the other datasets but covered between 58–80% of their expressomes per tissue. We therefore tested whether the different datasets were compatible and could be combined. Indeed, the overlaps between datasets in expressed genes per tissue were highly statistically significant in all cases (p-value<10^−97^, Fisher exact test). Moreover, corresponding tissues from the different datasets best correlated with each other in almost all cases (see Methods and Table S1). We also compared between the tissue distributions of genes per dataset and found them to be bi-modal, with most genes showing either tissue-specific or ubiquitous expression across tissues (Figure 2A). In particular, in the GNF dataset most genes were tissue-specific, but in the more recent RNA-seq and HPA datasets, in accordance with other expression datasets [4], most genes were ubiquitously expressed across all tissues. We observed similar bi-modal distributions upon using less stringent expression thresholds (Figure S1).

**Table 1:** A comparative view of tissue expressomes detected by each dataset individually and in combination.

Tissue	All^1^	GNF	HPA	RNA-seq	GNF/HPA % overlap^2^	GNF/RNA-seq % overlap^2^	HPA/RNA-seq % overlap^2^
Adipose	10,859	2,533	N/A	10,269	N/A	76.71	N/A
Adrenal	13,592	2,498	7,235	10,822	47.08	72.42	68.57
Brain	14,000	4,335	7,692	10,925	49.64	76.38	69.37
Breast	12,669	N/A	6,526	10,698	N/A	N/A	69.80
Colon	13,312	2,807	7,244	10,519	48.13	77.02	68.00
Heart	12,766	3,345	6,189	9,827	38.57	72.38	64.15
Kidney	13,662	2,025	7,672	10,945	47.56	73.23	69.81
Liver	11,958	2,531	6,202	8,842	40.42	70.88	58.98
Lung	13,853	3,010	7,465	11,063	45.85	75.68	69.86
Lymph Node	13,185	2,441	6,183	10,973	43.96	75.09	71.87
Ovary	12,918	1,567	5,111	11,165	35.48	73.77	72.28
Prostate	13,586	3,075	6,508	11,250	43.97	77.37	72.20
Skeletal Muscle	11736	1,751	5,805	8,851	37.64	66.08	58.05
Testis	148,19	3,176	7,744	12,567	49.78	80.29	76.86
Thyroid	13,518	3,360	6,982	10,938	47.02	78.96	70.60
White Blood Cells	10,844	5,750	N/A	9,466	N/A	76.03	N/A
**Median**	**13,248**	**2,807**	**6,754**	**10,873**	**45.85**	**75.68**	**69.81**

Numbers refer to proteins and protein-coding genes only.

1 The number of genes that are found to be expressed upon uniting the datasets.

2 The percent of overlap reflects the fraction of commonly-detected genes out of the smaller expressome.

To obtain an extensive view of the repertoire of expressed genes and their potential PPIs in each tissue we combined the three datasets. Specifically, we defined a gene as expressed in a tissue if it was found to be expressed in that tissue in at least one dataset. The resulting tissue expressomes maintained the bi-modal tissue distribution (Figure 2A): 61% of the genes were expressed in 14–16 tissues, henceforth denoted globally expressed genes, and 14% of the genes were expressed in 1–3 tissues, henceforth denoted tissue-specific genes. Testing for gene ontology (GO) enrichments we found that globally expressed genes were highly enriched for basic cellular processes common to living cells, such as RNA splicing (p<10^−28^) and protein transport (p<10^−26^, Table S2). Tissue-specific genes were enriched for tissue-specific processes such as spermatogenesis in testis (p = 7.3*10^−6^) and sensory perception in brain (p = 7.2*10^−4^, Table S3).

### Common features of tissue interactomes

To construct tissue interactomes we first gathered recent data of experimentally-detected human PPIs from four major public databases [16]–[19]. These data amounted to a global interactome consisting of 67,439 interactions between 11,225 proteins, thus covering 52% of the human protein-coding genes. We then constructed tissue interactomes by filtering the global interactome according to tissue expressomes (Figure 1). Specifically, each tissue interactome contained only those PPIs in which both interacting partners were found to be expressed in that tissue (see Methods). Each resulting tissue interactome covered more than half of the global human interactome, with 58–75% of the proteins and 63–79% of the PPIs. The tissue interactomes are provided at http://netbio.bgu.ac.il/tissueinteractoms.

An overall view of the tissue interactomes appears in Figure 2B. All the tissue interactomes shared a common core sub-network that contained 4,989 proteins and 26,370 PPIs. This core sub-network dominated all tissue interactomes by containing half or more of their proteins and PPIs. To test the consistency in expression levels of core proteins across tissues we applied the DESeq method [20]. Only 555 of the 4,989 core genes (11%) showed a significant change in expression (p-value≤0.01) in at least one tissue, implying that most core proteins are expressed at similar levels across tissues. As can be expected, core proteins were highly enriched for basic cellular processes (Table S4).

Another common feature of the tissue interactomes was the scale-free like distribution of their PPI degrees (degree is the number of interacting partners per protein). In each tissue most proteins had at most five interacting partners, while a small subset of proteins, denoted hubs, had over 45 interacting partners each (Figure S2). These 451 tissue hubs generally retained their high degree across tissues (Figure 2C), and were highly enriched for a variety of regulatory processes, such as regulation of transcription (36%, p<10^−15^) and regulation of signal transduction (18%, p<10^−12^, see Table S5). 221 of these tissue hubs were also found in the core sub-network and showed a similar regulatory nature relative to other core proteins (Table S6).

The wide range of PPI degrees led us to hypothesize that proteins with many interacting partners may require a larger number of molecules in order to support these interactions, relative to proteins with only a few interacting partners, as was previously observed in budding yeast [21]. We therefore correlated between PPI degrees and transcript level per gene in each tissue (see Methods). In all tissues these correlations were statistically significant (Figure 2D and Figure S3). These correlations were maintained despite the diversity across tissues in transcript levels and in PPI partners (Figure S4).

### A network view into transcript levels and tissue-specific PPIs of genes causing hereditary diseases

**Figure 3:**
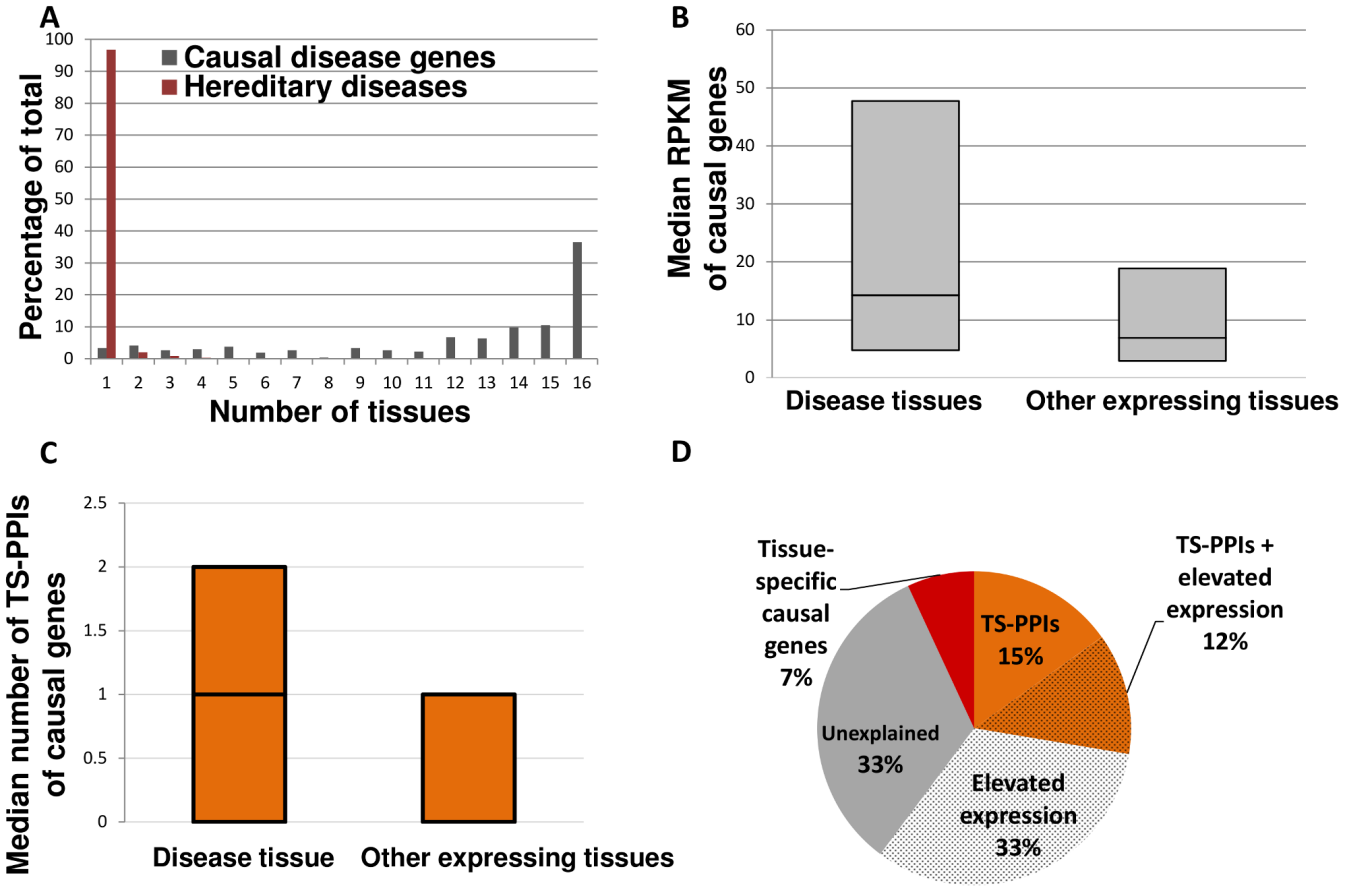
Tissue-related features of hereditary diseases and their causal genes. A. The tissue-distribution of hereditary diseases and their causal genes shows that diseases are manifested in few tissues, while most of their germline-aberrant causal genes are expressed in 10 tissues or more. The numbers of expressed causal genes across 1–16 tissues appear in Table S13. B. Causal genes tend to be more highly expressed in their disease tissues relative to other tissues in which they are expressed. We observed higher median expression levels in disease tissues for 128 out of the 203 germline-aberrant causal genes for which RPKM values were available (p-value<10^−4^). The box-plot diagram shows the quartiles (25%, 50% and 75%) of the median RPKM levels of causal genes; for each gene only tissues expressing the gene were considered. C. Causal genes involved in TS-PPI tend to have more TS-PPI in their disease tissues relative to other tissues. Out of 126 genes with TS-PPI, 58 genes had higher median TS-PPI in the disease tissue relative to non-disease tissues in which they are expressed (p-value<10^−4^). The box-plot diagram shows the quartiles (25%, 50% and 75%) of the median number of TS-PPI of causal genes, where for each gene only tissues expressing the gene were considered. The first (25%) and second (50%) quartiles of non-disease tissues were zero and therefore overlap with the X axis. D. The majority of the 303 hereditary diseases are associated with elevated expression and/or TS-PPIs of their causal genes in their disease tissues.

We gathered 303 hereditary diseases that manifested clinically in at least one of the 16 tissues that we analyzed, and their 233 causal germline-aberrant genes (see Methods and Figure S5). As shown in Figure 3A, most hereditary diseases manifested in a single tissue, and yet over 80% of their causal genes were expressed in 10 tissues or more. Thus, causal genes tend to elicit a clear phenotype in only a small subset of their expressing tissues.

We next tested whether the expression levels of causal genes differ between their disease- and unaffected tissues, as shown previously for the larger set of genetic diseases caused by somatic or germline aberrations [3]. To this end we compared between the median expression level of causal genes in their disease tissues and their median expression level in non-disease tissues (see Methods). We found that a significant fraction of these genes were expressed at elevated levels in their disease tissues (63%, randomization test p<10^−4^, Figure 3B), with almost a third of these having significantly high levels (28% with p≤0.01, DESeq analysis). Given the correlation between transcript levels and PPI degrees, we next tested whether causal genes also tend to have more PPIs in their disease tissue. This tendency too was significant (42%, randomization test p<10^−4^, Figure S6). Moreover, there was a significant overlap between causal genes with elevated expression levels and causal genes with higher PPI degrees in their disease tissue (Fisher exact test p = 0.02).

**Figure 4:**
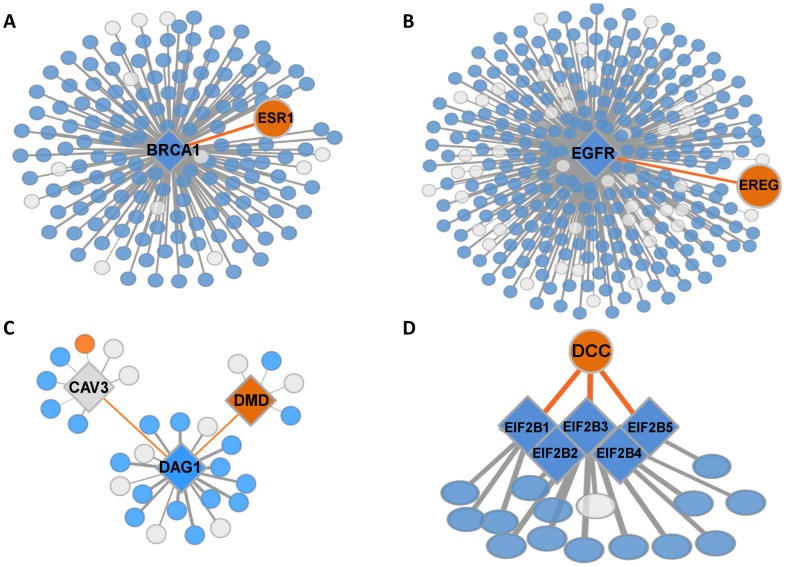
TS-PPIs illuminate disease-related tissue-specific effects of causal genes. Orange, blue and grey nodes denote tissue-specific, globally-expressed, and other proteins, respectively; diamond nodes mark hereditary disease genes; edges denote PPIs. A. BRCA1 is a globally-expressed tumor-suppressor hub, and ESR1 is an estrogen receptor protein that activates cellular proliferation. The breast-specific PPI linking BRCA1 and ESR1 provides a potential basis for the breast-specific effects of BRCA1 germline mutations [44]. B. A lung-specific PPI connects the widely-expressed epidermal growth factor receptor EGFR and its ligand protein epiregulin (EREG). Germline mutations in EGFR lead to lung cancer [30], and EREG was shown to confer invasive properties in an EGFR-dependent manner [31]. C. Muscle-specific PPIs connect the widely expressed trans-membrane cell adhesion receptor dystroglycan 1 (DAG1) to its muscle-specific ligand dystrophin (DMD), and to caveolin 3 (CAV3) which regulates DMD by preventing the DAG1-DMD PPI. Mutations in all three genes give rise to various forms of muscular dystrophies. D. The brain-specific PPIs that link members of the globally-expressed protein complex EIF2B to the netrin-1-receptor DCC may underlie the brain-specific effects of germline mutations in EIF2B complex members [35].

Given that causal genes tended to have more PPIs in their disease tissue, we tested whether they are also associated with PPIs that occur almost exclusively in that tissue. Such tissue-specific PPIs (TS-PPIs) can offer a clear molecular basis for the tissue-specific manifestation of hereditary diseases. Indeed, we found several examples where the TS-PPIs of causal genes in their disease tissues involved genes and interactions previously shown to be relevant for disease etiology (Table 2 and Figure 4, see Discussion). The full list of causal genes and their TS-PPIs appears in Table S8. We next turned to assess the prevalence of TS-PPIs among causal genes. We found that causal genes had a significantly higher tendency for TS-PPIs relative to interactome genes (Fisher exact test p = 8.8*10^−5^, Figure S7). Moreover, their TS-PPIs occurred preferentially in their disease tissues (randomization test p<10^−4^, Figure 3C). As could be expected, causal genes with more PPIs and causal genes with TS-PPIs in their disease tissue significantly overlapped (Fisher exact test p<10^−9^). However, there was no significant overlap between causal genes with TS-PPIs and causal genes with elevated transcript levels in their disease tissues (Fisher exact test p = 0.56). Thus, TS-PPIs and elevated transcript levels of causal genes in their disease tissues distinctly contribute to the emergence of tissue-specific phenotypes. As shown in Figure 3D, these two factors are related to 67% of the hereditary diseases in our dataset.

**Table 2:** Hereditary disease genes and their disease-related TS-PPI.

Disease	Causal gene (number of expressing tissues)	TS-PPI partner in disease tissue	Relation to disease
Familial hyper-cholesterolemia	LDLR (16)	PCSK9, liver	PCSK9 promotes LDLR degradation [45]
Androgen insensitivity	AR (10)	MAGEA11, testis	MAGEA11 increases AR activity [46]
Atrial septal defect 2	GATA4 (14)	NKX2-5, heart	NKX2-5 mutations are related to atrial septal defects [47]
Alzheimer disease type 3	PSEN1 (16)	ICAM5, brain	ICAM5 loss of function is related to dementia [48]
Alzheimer disease-4	PSEN2 (16)	ICAM5, brain	ICAM5 loss of function is related to dementia [48]
Alzheimer disease-4	PSEN2 (16)	GFAP, brain	GFAP splice variant expression is related to Alzheimer [49]
Dementia with Lewy Bodies (DLB)	SNCA (15)	SNCB, brain	SNCB mutations lead to DLB [50]
Dementia frontotemporal with or without parkinsonism	MAPT (13)	SLC1A2, brain	SLC1A2 polymorphisms are associated with essential tremor [51]
Sex Reversal	SOX9 (14)	NR5A1, testis	NR5A1 mutations are related to XY sex reversal [52]
Hereditary non-polyposis colorectal cancer Turcot syndrome	MLH1 (16)	MUC2, colon	Muc2 is involved in colorectal cancer suppression [53]
Muscular Dystrophy	DAG1 (16)	DMD, skeletal muscle	DMD is the ligand of DAG1 [32]
Muscular Dystrophy	DAG1 (16)	CAV3, skeletal muscle	This PPI regulates DMD recruitment to sarcolemma [33]
Hereditary Breast Cancer	BRCA1 (16)	ESR1, breast	BRCA1 inhibits ESR1 proliferative activity [29]
Lung Cancer	EGFR (15)	EREG, lung	EREG is EGFR's ligand, confers invasiveness [31]
Leukoencephalopathy with vanishing white matter	EIF2B1 complex proteins (14–16)	DCC, brain	DCC activates EIF2B translation complex [35]

## Discussion

The identification of germline-aberrant genes underlying many of the hereditary diseases provides an important step toward unraveling their molecular basis. Still, the remarkable tissue-specificity of hereditary diseases makes it clear that additional factors are governing disease manifestations. Relying on the utility of interactomes in understanding genotype-to-phenotype relationships [22], we applied here a comparative analysis of tissue interactomes to uncover determinants of the tissue-specificity of hereditary diseases.

We analyzed over 300 hereditary diseases and their causal genes. However, this set was limited by several factors: First, only diseases caused by mutations in protein-coding genes were included. Second, diseases had to be associated with at least one of the 16 tissues we analyzed. Given that the tissue associations were deduced by using a text-mining approach [3], these associations could be noisy or limited to the subset of hereditary diseases with clear tissue phenotypes. Third, the expression level of causal genes in their disease tissue had to reach a certain threshold, and thus diseases whose causal genes are lowly expressed might have been ignored. These limitations imply that our results may be more relevant for monogenic disorders with strong tissue phenotypes.

To construct tissue interactomes we combined three large-scale datasets of transcript or protein abundance across a multitude of tissues, which were obtained through three experimental techniques. We found relatively strong correlations between transcript levels in corresponding tissues, and statistically significant yet low correlations between transcript levels and protein abundance. The latter correlation was recently shown to be around 0.4 in simultaneous measurements from a common sample [23]. The lower correlations we observed likely stem from noisy estimates of transcript and protein abundance, and from correlating between measurements from different tissue samples.

Similarly to other studies of tissue interactomes (e.g., [9], [11]), we combined the datasets by associating a gene with a tissue if its expression in that tissue passed certain criteria in at least one dataset. This combination resulted in tissue expressomes that were unique in their extent (Table 1). At the same time, relying on no more than three sources allowed us to limit lab bias effects that would have been encountered upon analyzing a similar number of samples but from many different labs [24]. We then superimposed the subset of tissue-associated genes on the set of known PPIs, filtering out PPIs in which at least one interacting partner was not associated with the tissue. It is important to note that whether a PPI indeed occurs in the tissue depends on additional factors, such as the cellular localization of the interacting proteins and their posttranslational modifications. Nevertheless, expression of both partners is a necessary initial requirement, and therefore is often used as a filter for constructing tissue interactomes (e.g., [3], [7], [9]–[11]). The effectiveness of filtered tissue interactomes was demonstrated in two recent studies, which showed that they considerably improve the prioritization of disease genes relative to an unfiltered global interactome [10], [11].

The interactomes of the different tissues had common features. First, the majority of their genes were common to 14 or more interactomes (Figure 2). These globally expressed genes were enriched in basic cellular processes and formed a common core sub-network that dominated all tissue interactomes (Figure 3). Second, hubs in the different tissue interactomes were enriched in regulatory processes. Third, the different tissues shared significant correlations between transcript levels and PPI degrees (Figure 2D and Figure S3). Such correlation was previously observed in budding yeast [21] but not in human. One might assume that these correlations stem from the fact that PPIs between highly-expressed proteins are easier to detect. However, the detection of PPIs was often done outside of a human cell, through *in-vitro* assays or by using yeast cells (e.g. [25]). In such assays the transcript levels are unrelated to the *in-vivo* levels of these transcripts within human cells of different tissues, and therefore the bias toward genes with high transcript levels *in-vivo* is unlikely.

In view of these marked similarities between the different tissue interactomes, the hereditary diseases that we analyzed stood out as a critical manifestation of tissue-specificity. Contrary to what might be expected, only 7% of the tissue-specific hereditary diseases were associated with tissue-specific causal genes (Figure 3D). Instead, the large majority of causal genes were expressed in many tissues that, enigmatically, did not show marked disease phenotypes (Figure 3A). We next harnessed the tissue interactomes to identify features that distinguish causal genes in their disease tissues and could underlie the tissue-specific selectivity and vulnerability.

We found that causal genes tend to have elevated expression levels in their disease tissues relative to unaffected tissues in which they were expressed (Figure 3B). A similar tendency was observed previously among genes causal for genetic diseases excluding cancers [3]. The correlation we observed between transcript levels and PPI degrees (Figure 2D), and the law of mass-action that links gene dosage with probability of interactions [26], both suggest that causal genes will interact in a more promiscuous manner in their disease tissues. Indeed, we found that causal genes tend to have more PPIs in their disease tissues. Given that mutations leading to diseases were shown in some cases to disturb the physical interactions of disease proteins [27], [28], the higher tendency for potentially disturbed PPIs in disease tissues may underlie the increased vulnerability of these tissues.

The other feature that distinguishes causal genes in their disease tissues is their tendency for TS-PPIs, which is observed for 27% of the hereditary diseases (Figure 3D). Notably, such TS-PPIs can offer an explanation for the tissue-specific manifestation of a disease: while they may not comprise the entire disease mechanism, these interactions can enhance or propagate the aberrant phenotypes and thus contribute to the clinical manifestation (Table 2). An important implication of this observation relates to the interrogation of disease etiologies: Whereas current efforts to illuminate the molecular basis of diseases typically consider all the interactions involving causal genes in their disease tissues (e.g., [10], [11]), we suggest concentrating efforts on their TS-PPIs. We show in Table S8 that focusing on these TS-PPIs typically reduces the number of candidate PPIs by 8-fold, thus providing a powerful filter.

Below we demonstrate that TS-PPIs can highlight additional disease-related proteins and interactions effectively. Our first example relates to the widely-expressed tumor suppressor *BRCA1* that causes predisposition to hereditary breast and ovarian cancer. We found that, in breast, *BRCA1* is involved in a single TS-PPI, with the estrogen receptor *ESR1* that activates cell proliferation (Figure 4A). Indeed, it was previously demonstrated that through this interaction *BRCA1* inhibits *ESR1* and its proliferative activity in breast, and that this effect is reduced in mutated forms of BRCA1 [29]. The second example of a disease-related TS-PPI is that of the widely-expressed epidermal growth factor receptor EGFR. Germline and somatic mutations in EGFR lead to lung cancer [30]. Notably, we found that a lung-specific PPI connects EGFR to its ligand-protein epiregulin (EREG) that was shown to confer invasive properties in an EGFR-dependent manner [31]. Thus, the EGFR-EREG PPI has the potential to enhance the effect of EGFR aberration in a lung-specific manner (Figure 4B). The third example relates to aberrations in three genes that separately cause different subtypes of muscular dystrophy: dystroglycan 1 (DAG1), dystrophin (DMD), and caveolin 3 (CAV3). DMD is a muscle-specific protein that anchors the extracellular matrix to the cytoskeleton. It is also the ligand of DAG1, a globally expressed trans-membrane cell adhesion receptor that interacts with DMD in muscle only [32]. This interaction is prevented by another muscle-specific PPI, in which CAV3 binds to DAG1 [33]. These muscle-specific PPIs explain the muscle-specific phenotypes of DAG1 and CAV3 aberrations (Figure 4C). The last example is a putative explanation for leukoencephalopathy with vanishing white matter, a brain disease that manifests during childhood. The progressive white matter deterioration is caused by germline mutations in any of the five genes encoding the subunits of the translation initiation factor EIF2B, and cells harboring any of these mutations show decreased translation activity [34]. While EIF2B proteins are globally expressed, we found that they exhibit brain- and testis-specific interactions with the netrin-1-receptor DCC, which mediates axon guidance (Figure 4D). Notably, Tcherkezian et al. [35] that identified the relationships between DCC and EIF2B also showed that the absence of netrin significantly lowers cellular translation. We therefore propose that this relationship enhances the effect of EIF2B mutations in a brain-specific manner.

The distinct features we identified provide a starting point for elucidating the molecular basis of many hereditary diseases and can be further applied to filter the wealth of data being generated by large-scale disease-associations studies. In the future, additional tissue features could be considered, such as protein isoform concentrations [36] and relationships other than PPIs [37]. The comparative tissue analysis, along with the extensive resource of human tissue interactomes that we put forward, should become a standard framework for interpreting the wealth of disease-related data and for enhancing our understanding of the etiologies of hereditary diseases.

## Methods

### Expression data sources

GNF data [12] were downloaded from BioGPS [38], and all genes with intensity value above 100 in a tissue were considered as expressed [39]. HPA data [14] included proteins that were identified as expressed in a tissue, i.e., assigned as ‘low’, ‘medium’ or ‘high’ abundance based on manual assessment of tissue staining by antibodies against the proteins of interest. Proteins were further filtered by imposing stringent thresholds on the reliability and validity of their antibodies: When available, a medium or high antibody-reliability score was required; otherwise we required at least one supportive and no negative validity scores. In case of multiple measurements per tissue per gene we chose the highest value. RNA-seq data from Illumina Body Map 2.0 [15] were filtered for genes with at least 1 read per kilobase per million reads (RPKM). Results for a threshold of 0.3 RPKM were similar and appear in Figure S1. Analysis was limited to proteins and protein-coding genes only, and these were mapped to their Ensembl gene identifiers using BioMart [40]. Table S9 presents the numbers of genes and tissues measured in each dataset.

### Consolidation of expression and tissue data

Since RNA-seq data covered the largest number of genes per tissue we based our analysis on the 16 main human tissues profiled with RNA-seq. GNF and HPA each contained profiles for 15 and 14 of these tissues and their subparts, respectively. We manually consolidated the various tissue subparts according to the consolidation scheme given in Table S10. A gene was considered as expressed in a tissue if that gene or its protein product were found to be expressed in that tissue or the tissue subparts by at least one dataset. Compatibility among datasets was tested by (i) computing the overlaps in genes and interactions expressed per tissue using Fisher exact test, and (ii) computing the correlation in expression levels of commonly-expressed genes using Kendall's tau rank correlation, and ranking the correlations between matching tissues compared to correlations between non-matching tissues (Table S1). Figure S8 provides scatter plots comparing the expression levels of common genes in corresponding tissues measured by any two out of HPA, GNF and RNA-seq.

### PPI data sources

We assembled PPI data from BIOGRID [16], DIP [17], IntAct [18] and MINT [19]. Only experimentally-detected physical interactions were included, and their union formed the global human interactome.

### Construction of tissue interactomes

Tissue interactomes were constructed by filtering the PPIs in the global human interactome according to tissue expression data [41]. A PPI was included only if both pair-mates were found to be co-expressed in the same sample or in the same tissue subpart at the lowest hierarchy level (Table S10). PPIs from subparts of the same tissue were united to form the tissue interactome.

### Hubs

Interactome hubs were defined as those nodes in the network where the number of interacting partners (PPI degree) ranked among the top 5%. This resulted in a threshold of over 45 PPI partners for each interactome we analyzed.

### Statistical analysis

Pair-wise Kendall's tau rank correlations between datasets were computed for expression levels of commonly detected genes. GO enrichments were performed using DAVID [42]. The total number of human proteins was 21,450 according to BioMart [40]. Differential expression of genes in a tissue was computed using the DEseq method [20]. DESeq analysis was performed for 16 tissues, such that each run compared one tissue to all other 15 tissues. The statistical significance of the overlap between disease genes with elevated expression, higher PPI degree or TS-PPI was calculated using Fisher exact test while excluding tissue-specific disease genes.

### Correlation between RPKM levels and PPI degrees

In each tissue we computed the Spearman correlation between RPKM levels and PPI degrees of genes with RPKM readout above 0 and PPIs in the tissue. We also binned genes based on their RPKM levels into 10 equally-sized bins, and computed correlations using the median of each bin. In all tissues both types of correlations were highly statistically significant. The correlation values and figures appear in Figure S3.

### Disease-tissue-gene associations

Disease to tissue associations were taken from Lage et al. [3], and included manually curated associations or associations exceeding a cutoff of 15 yielding a precision of 85% as mentioned therein.

Using data from the OMIM database [1] we limited the set of diseases to include only non-somatic and allelic disorders, and extracted disease-genes that were causally associated with those diseases. Data of hereditary cancers were downloaded from the cancer gene census website [43], was limited to cancer germline mutation genes, and manually associated with disease tissues. Only diseases that were associated with at least one of the 16 main tissues and whose causal disease gene was expressed in that tissue were analyzed.

### Comparative analysis of RPKM levels of disease genes

We compared the RPKM values of a disease gene between its disease and non-disease tissues in two ways. To identify elevated expression we compared between (i) the median RPKM of that gene in its disease tissues, and (ii) the median RPKM of that gene in its non-disease tissues. The permutation test used to assess the significance of the results is described in the following subsection. To identify significant over-expression we used the DEseq method as described in the ‘statistical analysis’ subsection above [20]. The subset of tissues considered for a specific gene was limited to tissues in which the gene was indeed expressed.

### Statistical significance of features of disease genes in their disease tissues relative to non-disease tissues

We used a permutation test to assess the statistical significance of the number of genes whose median value (RPKM level, PPI degree or number of TS-PPI) in their disease tissues was higher than the corresponding median value in their non-disease tissues. Specifically, for each disease gene we randomly selected a set of *x* disease tissues out of the set of tissues expressing that gene, where *x* was set to the number of original disease tissues for that gene. The remaining, non-selected tissues expressing the gene were considered as the gene's random non-disease tissues. In all calculations we ignored tissue-specific disease genes as their set of non-disease tissues could be empty. We then computed the relevant median value for that gene in its randomly-selected disease tissues (median value denoted V_d) and the median value in its random non-disease tissues (median value denoted V_nd). If the median in disease tissues was higher (i.e., V_d>V_nd) the gene was considered as success in the permutation test. We applied this test to all disease genes and counted the total number of random successes in that run. We repeated this analysis 10,000 times. We computed the p-value as the fraction of runs out of the 10,000 runs in which the total number of random successes was at least as high as the number of successes in the original data.

### Additional data files

The supplementary material file contains Figures S1, S2, S3, S4, S5, S6, S7, S8 and Tables S1, S2, S3, S4, S5, S6, S7, S8, S9, S10, S11, S12, S13. The tissue interactomes can be found at http://netbio.bgu.ac.il/tissueinteractoms.

## Supporting Information

Figure S1 **The distribution of genes by number of expressing tissues remains bi-modal when gene expression thresholds are relaxed.** Most genes are either globally expressed or tissue-specific, yet the tendency for global expression is enhanced. GNF refers to the study of Su et al (1). HPA refers to data of the human protein atlas (2). RNA-seq refers to RNA-sequencing data (3). GNF genes were considered as expressed in a tissue if their intensity value was above 30, and RNA-seq genes were considered as expressed in a tissue if their RPKM was at least 0.3. Total is the combined expression.
(PDF)

Figure S2 **The 16 tissue interactomes show similar distributions of PPI degrees.** The PPI degree of a protein in a tissue is the number of its PPI partners in that tissue. Most genes have at most 5 PPI partners in a tissue. Also shown are the PPI degree distributions within the global interactome and within the backbone interactome shared by all tissues.
(PDF)

Figure S3 **Gene expression levels and their PPI degrees are correlated in all 16 tissues.** A. Binned expression data: Each box-plot diagram shows the quartiles (25%, 50% and 75%) of the sorted PPI-degree values (Y axis) in each RPKM bin (X axis). The Spearman correlations of the median values were above 0.91 and statistically significant (p<2.4*10^−4^) in all tissues. B. No binning of expression data: Each scatter plot shows the log2 RPKM values (X-axis) and the PPI-degree values (Y axis) for a specific tissue. The Spearman correlations were above 0.2 for 14 out of 16 tissues and statistically significant (p<2.97*10^−18^) in all tissues. Correlations and p-values for each tissue were as follows: Adipose 0.23, p<8.13*10^−80^ ; Adrenal 0.23 p<9.65*10^−87^; Brain 0.22 p<1.57*10-^78^; Breast 0.20, p<7.47*10^−62^; Colon 0.22, p<7.07*10-^76^; Heart 0.18, p<2.46*10^−49^; Kidney 0.19, p<1.61*10^−60^; Liver 0.11, p<2.97*10^−18^; Lung 0.22, p<2.41*10^−79^; Lymph Node 0.23, p<8.17*10^−89^; Skeletal Muscle 0.21, p<1.91*10^−63^; Ovary 0.25, p<4.57*10^−105^; Prostate 0.22, p<5.09*10^−83^; Testis 0.25, p<9.19*10^−109^; Thyroid 0.23, p<5.72*10^−88^; WBC 0.20, p<2.65*10^−3^.
(PDF)

Figure S4 **The diversity across tissues in transcript levels and in genes' PPI partners.** A. A heat map showing the diversity in RPKM expression levels. Each row represents a gene *(g)*, each column represents a tissue *(t)*, and each entry *(g,t)* represents the rank of the RPKM level of the gene *g* in tissue *t*. The ranking is in decreasing order of expression, such that the most highly expressed gene is ranked 1. The ranking is from blue to gray, white entries represent non-expressing tissues. B. The diversity on PPI partners of genes across tissues. Each row represents a gene and the percentage of its PPI partners that are expressed in 1–3 tissues (orange), 4–13 tissues (blue) and 14–16 tissues (grey).
(PDF)

Figure S5 **Tissue-association of hereditary diseases, their causally-associated hereditary disease genes, and the disease-to-gene associations per tissue.** All tissues manifest at least one hereditary disease.
(PDF)

Figure S6 **The PPI degrees of causal genes across tissues.** A. The PPI degree distribution of causal genes across tissues is scale-free like. Similarly to other interactome genes, most causal genes have at most five PPI partners. B. Causal genes tend to have a higher PPI degree in their disease tissues relative to other tissues (42%, randomization test p<10^−4^). The box-plot diagram shows the quartiles (25%, 50% and 75%) of the median PPI degree of causal genes; for each gene only tissues expressing the gene were considered.
(PDF)

Figure S7 **Causal genes are significantly enriched in genes involved in tissue-specific PPIs.** Each pie chart describes the fraction of genes with 0 to 5 and above tissue-specific PPIs. Notably, 54% of the causal genes have at least one tissue-specific PPI, relative to only 42% of all interactome genes (p = 8.8*10^−5^, Fisher exact test).
(PDF)

Figure S8 **Scatter plots comparing the expression levels of genes measured in corresponding tissues by any two methods out of HPA, GNF and RNA-seq.** The poor correlations observed for HPA stem from the qualitative nature of protein abundance measurements (proteins abundance is either ‘low’, ‘medium’ or ‘high’ and determined based on manual assessment of antibody staining), while gene expression levels nicely correlated despite differences in samples and technique. Top panel: HPA vs. GNF r = 0.085, p = 1.51e-59. Middle panel: HPA vs. RNA-seq r = 0.085, p = 6.62e-233. Bottom panel: GNF vs. RNA-Seq r = 0.32, p = 0.0. All correlations were measured using Kendall's tau rank correlation.
(PDF)

Table S1 **Compatibility between corresponding tissues from different datasets.** Table S1A. Overlap in edges between corresponding tissues shows significant overlap between different datasets. Table S1B. Correlations between corresponding tissues from different datasets as determined by the expression levels of commonly detected genes in each tissue.
(PDF)

Table S2 **Gene ontology (GO) enrichment of globally expressed genes relative to all expressed genes.**
(PDF)

Table S3 **GO enrichment of tissue-specific genes relative to all expressed genes.**
(PDF)

Table S4 **GO enrichment of backbone proteins relative to all interactome proteins.**
(PDF)

Table S5 **GO enrichment of tissue hubs versus the global interactome.**
(PDF)

Table S6 **GO enrichment of global hubs versus global genes.**
(PDF)

Table S7 **Distribution of the number of hereditary diseases and their causal germline-aberrant disease genes by number of disease tissues they affect shows that most hereditary diseases are tissue-specific.**
(PDF)

Table S8 **Tissue-selective hereditary diseases and their tissue-specific PPIs in their disease tissues.**
(PDF)

Table S9 **Overview of the numbers of genes and tissues measured in each dataset.**
(PDF)

Table S10 **Consolidation of tissues from the different datasets into 16 main tissues.**
(PDF)

Table S11 **The distribution of the number of PPIs across 1–16 tissues.**
(PDF)

Table S12 **The numbers of genes and PPIs in the interactome of each tissue.**
(PDF)

Table S13 **The distribution of the number of expressed causal genes across 1–16 tissues.**
(PDF)
