# Supplementary material for: Comparative Analysis of Human Tissue Interactomes Reveals Factors Leading to Tissue-Specific Manifestation of Hereditary Diseases
Source: PLoS Comput Biol. 2014 Jun 12;10(6):e1003632. doi: 10.1371/journal.pcbi.1003632 (PMC4055280; doi:10.1371/journal.pcbi.1003632)

Figure S1: The distribution of genes by number of expressing tissues remains bi-modal when gene expression thresholds are relaxed. Most genes are either globally expressed or tissue-specific, yet the tendency for global expression is enhanced. GNF refers to the study of Su AI et al., PNAS 2004. HPA refers to data of the human protein atlas (Berglund L. et al., Mol Cell Proteomics 2008). RNA-seq refers to RNA-sequencing data (Bradley RK et al, PLoS Biol 2012). GNF genes were considered as expressed in a tissue if their intensity value was above 30, and RNA-seq genes were considered as expressed in a tissue if their RPKM was at least 0.3. Total is the combined expression dataset.

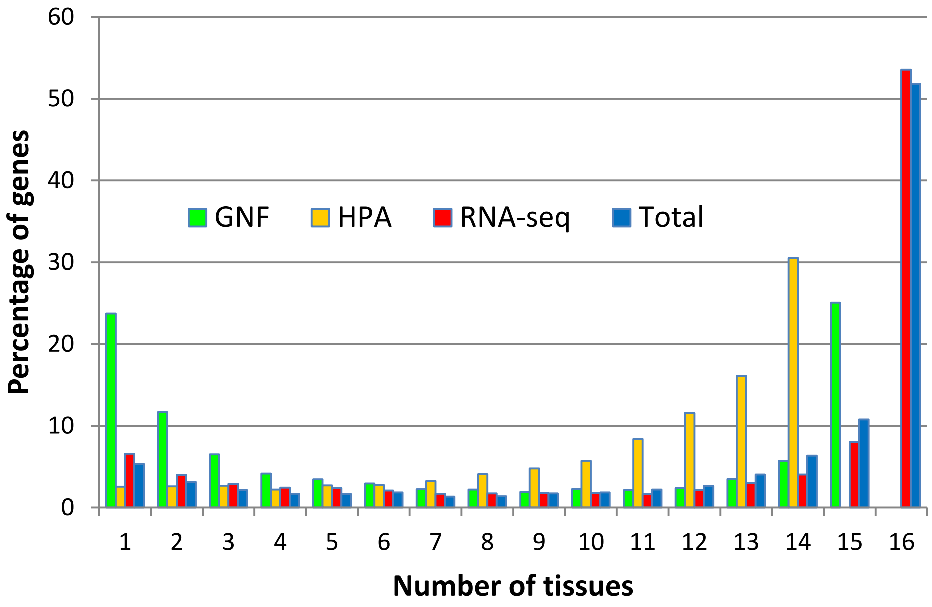

Supplement: Figure S1 — The distribution of genes by number of expressing tissues remains bi-modal when gene expression thresholds are relaxed. Most genes are either globally expressed or tissue-specific, yet the tendency for global expression is enhanced. GNF refers to the study of Su et al (1). HPA refers to data of the human protein atlas (2). RNA-seq refers to RNA-sequencing data (3). GNF genes were considered as expressed in a tissue if their intensity value was above 30, and RNA-seq genes were considered as expressed in a tissue if their RPKM was at least 0.3. Total is the combined expression. (PDF) [file pcbi.1003632.s001.pdf]
