# Supplementary material for: Comparative Analysis of Human Tissue Interactomes Reveals Factors Leading to Tissue-Specific Manifestation of Hereditary Diseases
Source: PLoS Comput Biol. 2014 Jun 12;10(6):e1003632. doi: 10.1371/journal.pcbi.1003632 (PMC4055280; doi:10.1371/journal.pcbi.1003632)

Figure S3: Transcript levels and PPI degrees are correlated in all 16 tissues.

A. Binned expression data: Each box-plot diagram shows the quartiles (25%, 50% and 75%) of the sorted PPI-degree values (Y axis) in each RPKM bin (X axis). The Spearman correlations of the median values were above 0.91 and statistically significant ($p<2.4*10^{-4}$) in all tissues.

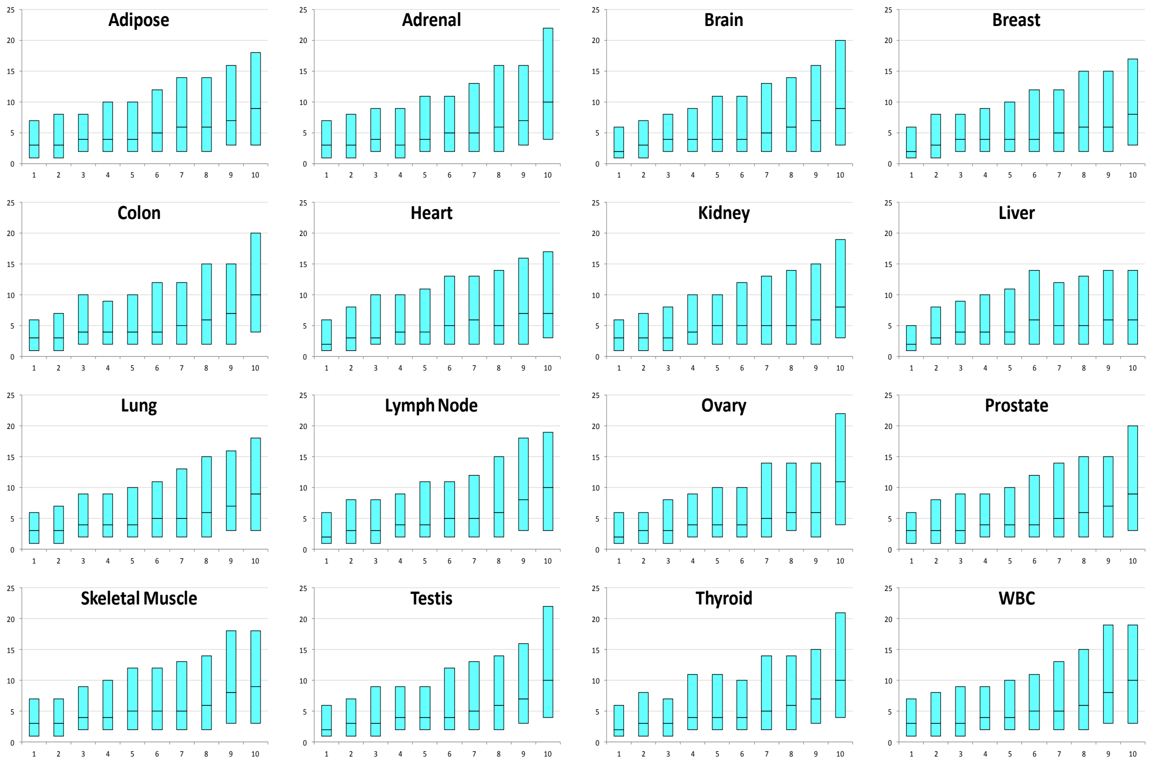

B. No binning of expression data: Each scatter plot shows the log2 RPKM values (X-axis) and the PPI-degree values (Y axis) for a specific tissue. The Spearman correlations were above 0.2 for 14 out of 16 tissues and statistically significant ($p<2.97*10^{-18}$) in all tissues. Correlations and p-values for each tissue were as follows:

Adipose 0.23, $p<8.13*10^{-80}$; Adrenal 0.23 $p<9.65*10^{-87}$; Brain 0.22 $p<1.57*10^{-78}$; Breast 0.20, $p<7.47*10^{-62}$; Colon 0.22, $p<7.07*10^{-76}$; Heart 0.18, $p<2.46*10^{-49}$; Kidney 0.19, $p<1.61*10^{-60}$; Liver 0.11, $p<2.97*10^{-18}$; Lung 0.22, $p<2.41*10^{-79}$; Lymph Node 0.23, $p<8.17*10^{-89}$; Skeletal Muscle 0.21, $p<1.91*10^{-63}$; Ovary 0.25, $p<4.57*10^{-105}$; Prostate 0.22, $p<5.09*10^{-83}$; Testis 0.25, $p<9.19*10^{-109}$; Thyroid 0.23, $p<5.72*10^{-88}$; WBC 0.20, $p<2.65*10^{-30}$**.**

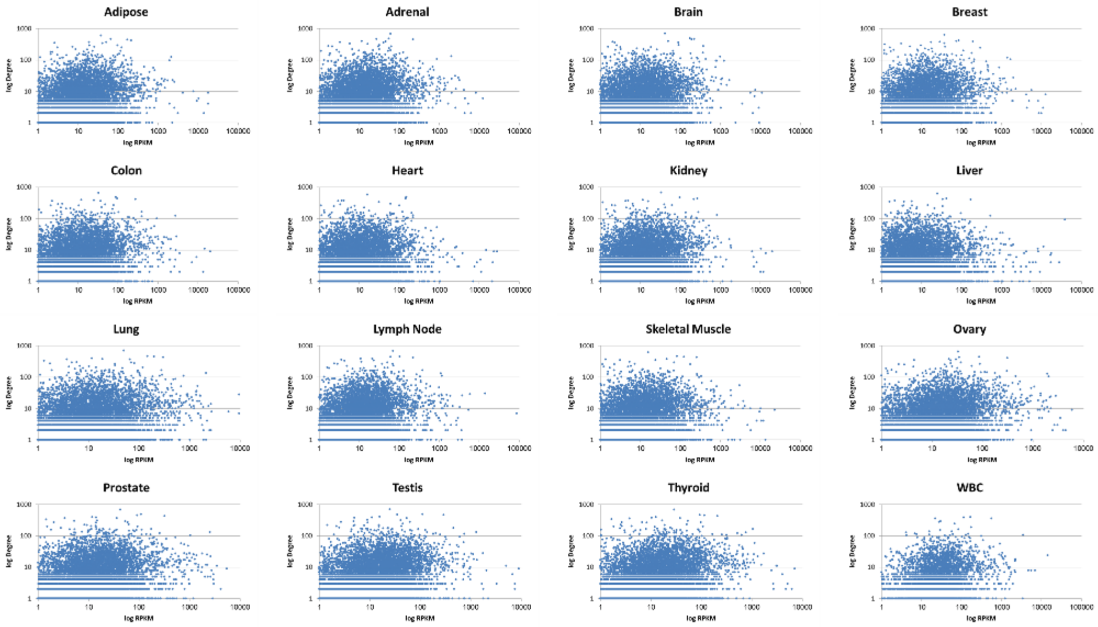

Supplement: Figure S3 — Gene expression levels and their PPI degrees are correlated in all 16 tissues. A. Binned expression data: Each box-plot diagram shows the quartiles (25%, 50% and 75%) of the sorted PPI-degree values (Y axis) in each RPKM bin (X axis). The Spearman correlations of the median values were above 0.91 and statistically significant (p<2.4*10−4) in all tissues. B. No binning of expression data: Each scatter plot shows the log2 RPKM values (X-axis) and the PPI-degree values (Y axis) for a specific tissue. The Spearman correlations were above 0.2 for 14 out of 16 tissues and statistically significant (p<2.97*10−18) in all tissues. Correlations and p-values for each tissue were as follows: Adipose 0.23, p<8.13*10−80 ; Adrenal 0.23 p<9.65*10−87; Brain 0.22 p<1.57*10-78; Breast 0.20, p<7.47*10−62; Colon 0.22, p<7.07*10-76; Heart 0.18, p<2.46*10−49; Kidney 0.19, p<1.61*10−60; Liver 0.11, p<2.97*10−18; Lung 0.22, p<2.41*10−79; Lymph Node 0.23, p<8.17*10−89; Skeletal Muscle 0.21, p<1.91*10−63; Ovary 0.25, p<4.57*10−105; Prostate 0.22, p<5.09*10−83; Testis 0.25, p<9.19*10−109; Thyroid 0.23, p<5.72*10−88; WBC 0.20, p<2.65*10−3. (PDF) [file pcbi.1003632.s003.pdf]
