# Supplementary material for: Comparative Analysis of Human Tissue Interactomes Reveals Factors Leading to Tissue-Specific Manifestation of Hereditary Diseases
Source: PLoS Comput Biol. 2014 Jun 12;10(6):e1003632. doi: 10.1371/journal.pcbi.1003632 (PMC4055280; doi:10.1371/journal.pcbi.1003632)

Figure S4: The diversity across tissues in transcript levels and in genes' PPI partners.

A. A heat map showing the diversity in RPKM expression levels. Each row represents a gene *(g)*, each column represents a tissue *(t)*, and each entry *(g,t)* represents the rank of the RPKM level of the gene *g* in tissue *t*. The ranking is in decreasing order of expression, such that the most highly expressed gene is ranked 1. The ranking is from blue to gray, white entries represent non-expressing tissues.

B. The diversity in PPI partners of genes across tissues. Each row represents a gene and the percentage of its PPI partners that are expressed in 1-3 tissues (orange), 4-13 tissues (blue) and 14-16 tissues (grey).

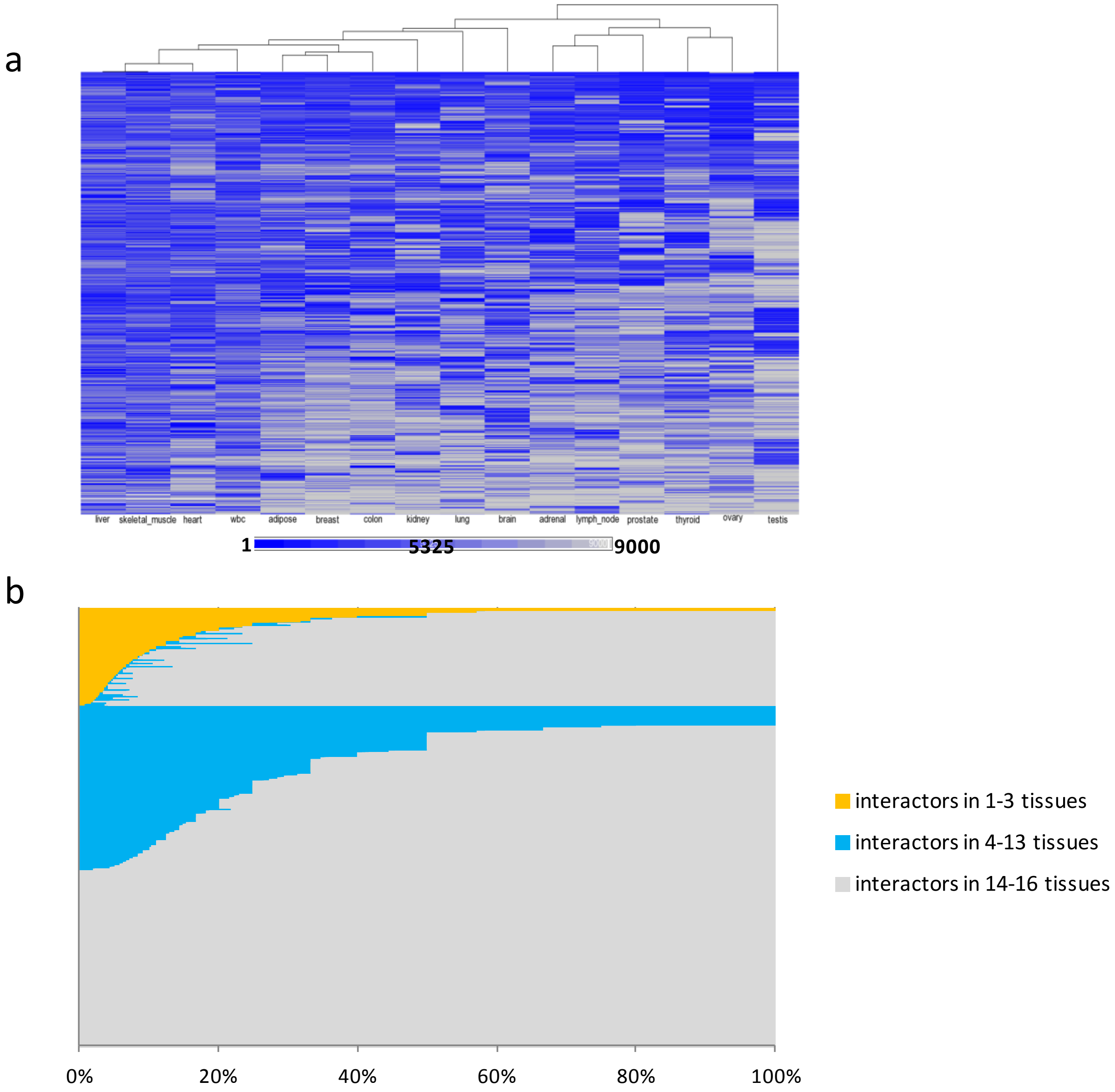

Supplement: Figure S4 — The diversity across tissues in transcript levels and in genes' PPI partners. A. A heat map showing the diversity in RPKM expression levels. Each row represents a gene (g), each column represents a tissue (t), and each entry (g,t) represents the rank of the RPKM level of the gene g in tissue t. The ranking is in decreasing order of expression, such that the most highly expressed gene is ranked 1. The ranking is from blue to gray, white entries represent non-expressing tissues. B. The diversity on PPI partners of genes across tissues. Each row represents a gene and the percentage of its PPI partners that are expressed in 1–3 tissues (orange), 4–13 tissues (blue) and 14–16 tissues (grey). (PDF) [file pcbi.1003632.s004.pdf]
