# Supplementary material for: Comparative Analysis of Human Tissue Interactomes Reveals Factors Leading to Tissue-Specific Manifestation of Hereditary Diseases
Source: PLoS Comput Biol. 2014 Jun 12;10(6):e1003632. doi: 10.1371/journal.pcbi.1003632 (PMC4055280; doi:10.1371/journal.pcbi.1003632)

Figure S5: Tissue-association of hereditary diseases, their causal genes, and the disease-to-gene associations per tissue. All tissues manifest at least one hereditary disease.

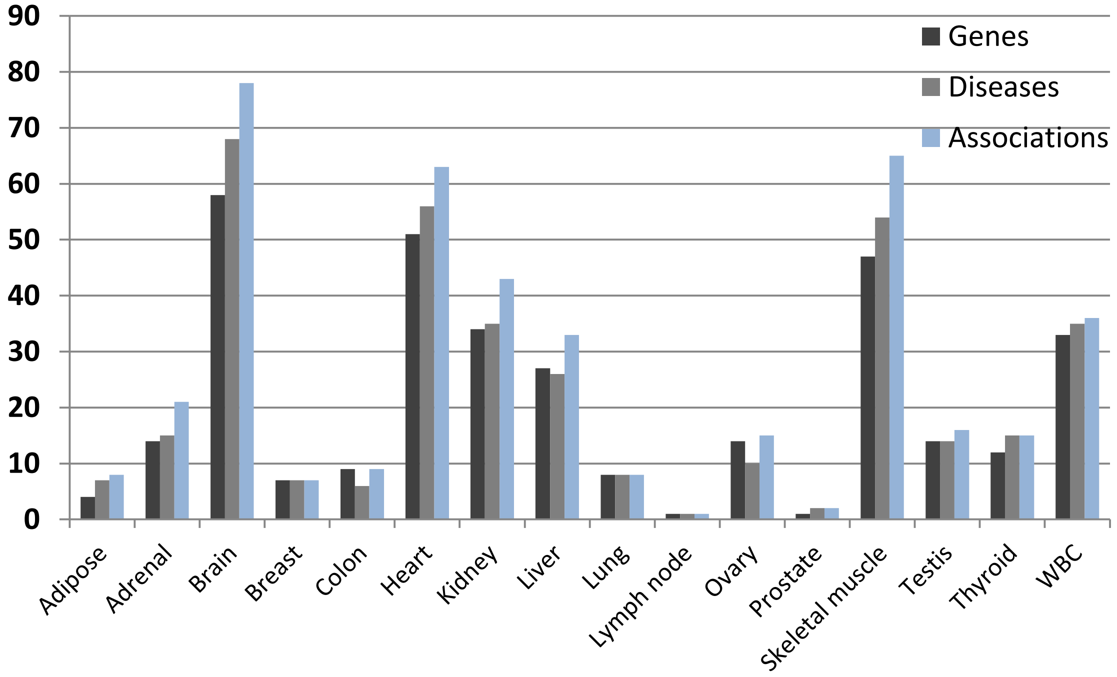

Supplement: Figure S5 — Tissue-association of hereditary diseases, their causally-associated hereditary disease genes, and the disease-to-gene associations per tissue. All tissues manifest at least one hereditary disease. (PDF) [file pcbi.1003632.s005.pdf]
