# Supplementary material for: Comparative Analysis of Human Tissue Interactomes Reveals Factors Leading to Tissue-Specific Manifestation of Hereditary Diseases
Source: PLoS Comput Biol. 2014 Jun 12;10(6):e1003632. doi: 10.1371/journal.pcbi.1003632 (PMC4055280; doi:10.1371/journal.pcbi.1003632)

Figure S6: The PPI degrees of causal genes across tissues.

A. The PPI degree distribution of causal genes across tissues is scale-free like. Similarly to other interactome genes, most causal genes have at most five PPI partners.

B. Causal genes tend to have higher PPI degree in their disease tissues relative to other tissues (42%, randomization test $p<10^{-4}$). The box-plot diagram shows the quartiles (25%, 50% and 75%) of the median PPI degree of disease genes; for each gene only tissues expressing the gene were considered.

A

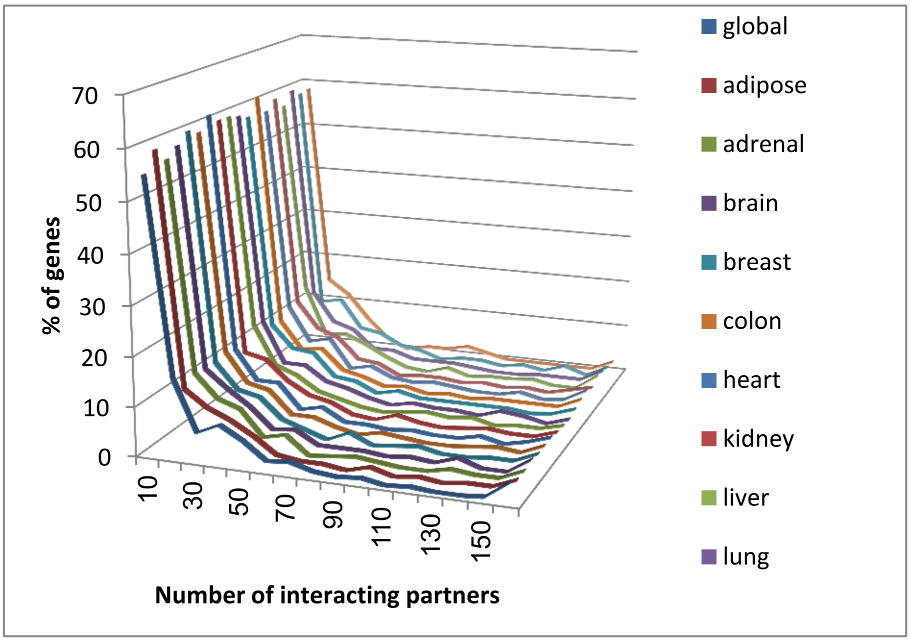

B

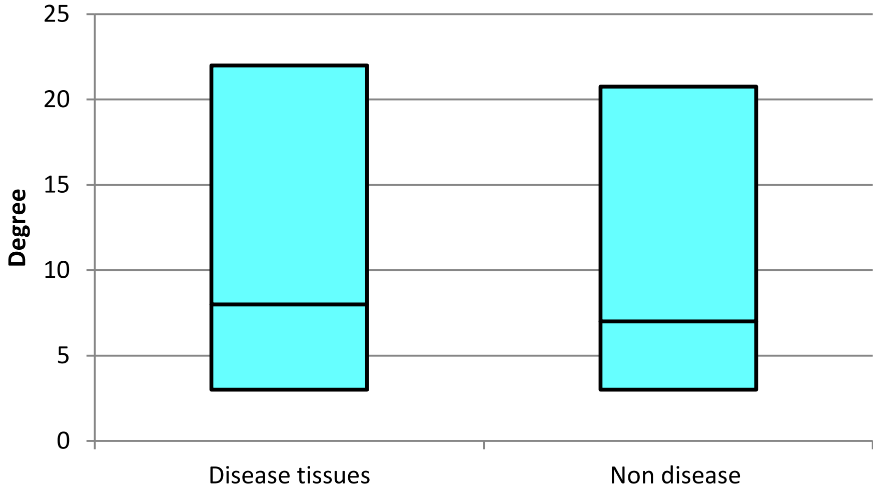

Supplement: Figure S6 — The PPI degrees of causal genes across tissues. A. The PPI degree distribution of causal genes across tissues is scale-free like. Similarly to other interactome genes, most causal genes have at most five PPI partners. B. Causal genes tend to have a higher PPI degree in their disease tissues relative to other tissues (42%, randomization test p<10−4). The box-plot diagram shows the quartiles (25%, 50% and 75%) of the median PPI degree of causal genes; for each gene only tissues expressing the gene were considered. (PDF) [file pcbi.1003632.s006.pdf]
