# Supplementary material for: Comparative Analysis of Human Tissue Interactomes Reveals Factors Leading to Tissue-Specific Manifestation of Hereditary Diseases
Source: PLoS Comput Biol. 2014 Jun 12;10(6):e1003632. doi: 10.1371/journal.pcbi.1003632 (PMC4055280; doi:10.1371/journal.pcbi.1003632)

Figure S7: Causal genes are significantly enriched in genes involved in tissue-specific PPIs. Each pie chart describes the fraction of genes with tissue-specific PPI numbers ranging from 0 to 5 and above. Notably, 54% of the hereditary disease genes have at least one tissue-specific PPI, relative to only 42% of all interactome genes ($p=8.8*10^{-5}$, Fisher exact test).

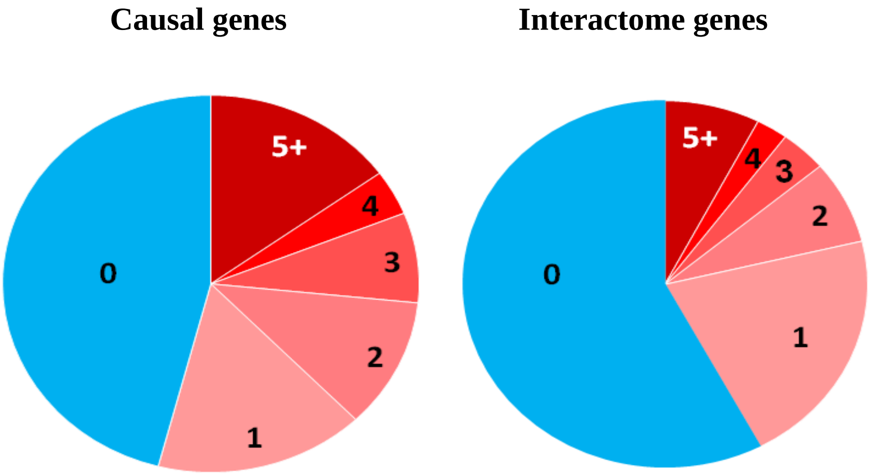

Supplement: Figure S7 — Causal genes are significantly enriched in genes involved in tissue-specific PPIs. Each pie chart describes the fraction of genes with 0 to 5 and above tissue-specific PPIs. Notably, 54% of the causal genes have at least one tissue-specific PPI, relative to only 42% of all interactome genes (p = 8.8*10−5, Fisher exact test). (PDF) [file pcbi.1003632.s007.pdf]
