# Supplementary material for: Comparative Analysis of Human Tissue Interactomes Reveals Factors Leading to Tissue-Specific Manifestation of Hereditary Diseases
Source: PLoS Comput Biol. 2014 Jun 12;10(6):e1003632. doi: 10.1371/journal.pcbi.1003632 (PMC4055280; doi:10.1371/journal.pcbi.1003632)

Figure S8: Scatter plots comparing the expression levels of genes measured in corresponding tissues by any two methods out of HPA, GNF and RNA-seq. Top panel: HPA vs. GNF r=0.085, p=1.51e-59. Middle panel: HPA vs. RNA-seq r=0.085, p=6.62e-233. Bottom panel: GNF vs. RNA-Seq r=0.32, p=0.0. All correlations were measured using Kendall's tau rank correlation. Transcript levels correlated despite differences in samples and technique (bottom panel). The poor correlations observed for HPA may stem from the qualitative nature of protein abundance measurements (proteins abundance is either 'low', 'medium' or 'high' and determined based on manual assessment of antibody staining).

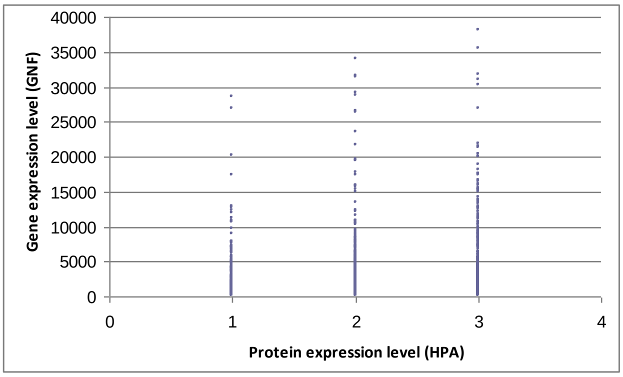

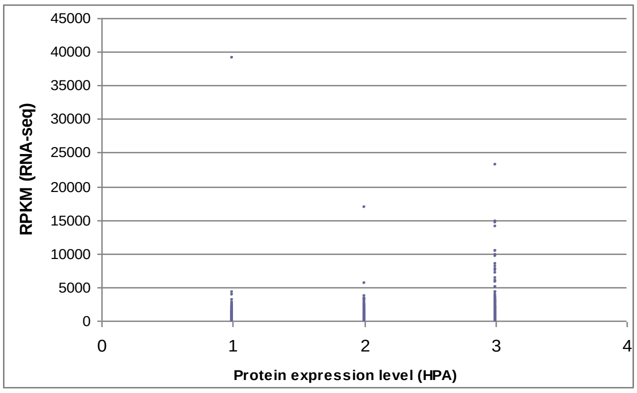

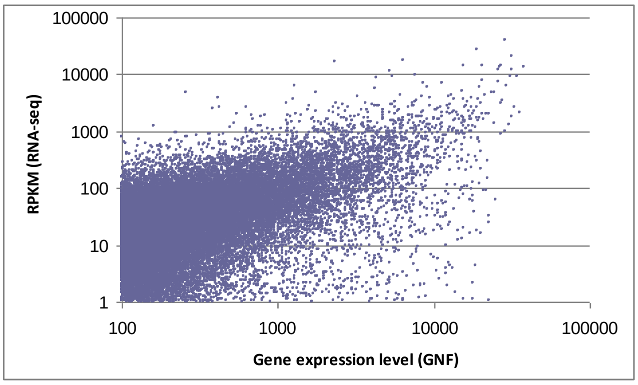

Supplement: Figure S8 — Scatter plots comparing the expression levels of genes measured in corresponding tissues by any two methods out of HPA, GNF and RNA-seq. The poor correlations observed for HPA stem from the qualitative nature of protein abundance measurements (proteins abundance is either ‘low’, ‘medium’ or ‘high’ and determined based on manual assessment of antibody staining), while gene expression levels nicely correlated despite differences in samples and technique. Top panel: HPA vs. GNF r = 0.085, p = 1.51e-59. Middle panel: HPA vs. RNA-seq r = 0.085, p = 6.62e-233. Bottom panel: GNF vs. RNA-Seq r = 0.32, p = 0.0. All correlations were measured using Kendall's tau rank correlation. (PDF) [file pcbi.1003632.s008.pdf]
