# Supplementary material for: Comparative Analysis of Human Tissue Interactomes Reveals Factors Leading to Tissue-Specific Manifestation of Hereditary Diseases
Source: PLoS Comput Biol. 2014 Jun 12;10(6):e1003632. doi: 10.1371/journal.pcbi.1003632 (PMC4055280; doi:10.1371/journal.pcbi.1003632)
Supplement: Table S1 — Compatibility between corresponding tissues from different datasets. Table S1A. Overlap in edges between corresponding tissues shows significant overlap between different datasets. Table S1B. Correlations between corresponding tissues from different datasets as determined by the expression levels of commonly detected genes in each tissue. (PDF) [file pcbi.1003632.s009.pdf]

Table S1: Compatibility between matching tissues from different datasets.

Table S1A. Correlations between corresponding tissues from different sources as determined by the expression levels of commonly detected genes in each tissue pair.

Expression data	Tissue	Rank[1]	Kendall's tau correlation	P-value
Transcript levels (GNF: RNA-seq)[2]	Adipose	1	0.36	9.5e-130
	Adrenal	2	0.26	3.12e-64
	Brain	1	0.34	4.2e-225
	Colon	1	0.35	5.71e-132
	Heart	1	0.32	1.09e-126
	Kidney	1	0.33	3.33e-82
	Liver	1	0.39	8.56e-136
	Lung	1	0.37	1.38e-157
	Lymph Node	1	0.35	4.66e-113
	Ovary	1	0.31	8.95e-58
	Prostate	1	0.31	2.44e-120
	Skeletal Muscle	1	0.3	2.92e-55
	Testis	1	0.35	1.78e-161
	Thyroid	1	0.36	2.45e-174
	WBC	1	0.37	1.98e-297
Protein and gene expression levels (HPA: GNF or RNA-seq)[3]	Adrenal	1	0.07	9.72e-05
	Brain	1	0.12	3.57e-44
	Breast	3	0.07	1.92e-11
	Colon	1	0..06	0.00061829
	Heart	1	0.07	2.14e-11
	Kidney	1	0.106	7.89e-07
	Liver	1	0.11	3.69e-24
	Lung	1	0.09	2.24e-24
	Lymph Node	1	0.11	1.97e-08
	Ovary	1	0.09	8.31e-18
	Prostate	1	0.12	4.74e-11
	Skeletal Muscle	1	0.03	0.002
	Testis	1	0.13	9.36e-51
	Thyroid	1	0.09	2.75e-23

[1]The ranking of the correlation value of corresponding tissues (e.g. Adipose in GNF versus Adipose in RNA-seq) compared to non-corresponding tissues (e.g. Adipose in GNF versus Colon in RNA-seq). Highest correlation value is ranked 1.

[2] GNF tissues best correlated with their corresponding RNA-seq tissues in 14 of the 15 tissues that were measured by both GNF and RNA-seq.

[3] HPA tissues best correlated with their corresponding gene tissues in 13 of the 14 tissues for which protein expression levels were measured (best rank shown).

Table S1B. Overlap in edges between matching tissues shows significant overlap between different sources.

Tissue	GNF Edges	HPA Edges	RNA-Seq edges	GNF/RNA-Seq %overlap[1]	P value[2]	HPA/RNA-Seq %overlap [1]	P value
Adipose	5176	N/A	36291	70.92	4.8e-151	N/A	
Adrenal	4869	18499	38036	68.12	8.7e-68	71.97	0
Brain	11202	20557	37122	70.51	8.4e-294	70.89	0
Breast	N/A	18042	37624	N/A	N/A	70.82	0
Colon	6417	19680	37002	68.87	3.8e-128	70.71	0
Heart	7684	11811	34379	67.28	7.3e-207	66.90	4.9e-324
Kidney	3112	21025	37657	65.16	1.4e-27	71.42	0
Liver	4470	14176	30416	65.32	2.7e-174	59.40	9.9e-324
Lung	6779	20288	38480	66.98	1.1e-69	71.72	0
Lymph Node	5057	16759	39057	69.48	1.1e-69	73.5	0
Ovary	2116	12454	38511	68.80	1.8e-29	73.12	0
Prostate	7220	16888	39029	68.78	1.5e-90	72.73	0
Skeletal Muscle	2523	11400	31616	64.96	1.8e-77	62.48	1.1e-294
Testis	7242	21232	41173	72.46	6.0e-103	75.54	0
Thyroid	8096	17881	38193	71.22	4.0e-182	73.21	0
WBC	20628	N/A	33725	68.76	0	N/A	N/A

[1] Percent is based on fraction of overlap out of the smaller set.

[2] Computed using Fisher exact test.
