# Supplementary material for: Comparative Analysis of Human Tissue Interactomes Reveals Factors Leading to Tissue-Specific Manifestation of Hereditary Diseases
Source: PLoS Comput Biol. 2014 Jun 12;10(6):e1003632. doi: 10.1371/journal.pcbi.1003632 (PMC4055280; doi:10.1371/journal.pcbi.1003632)
Supplement: Table S2 — Gene ontology (GO) enrichment of globally expressed genes relative to all expressed genes. (PDF) [file pcbi.1003632.s010.pdf]

Table S2: Gene ontology (GO) enrichment of globally expressed genes relative to all expressed genes. Analysis was performed using the DAVID functional annotation chart with GO term Level of 5. Only terms with a corrected p-value < 10^{-5} with at least 50 genes in the query set are reported. DAVID identified 7,004 genes out of 10,490 globally expressed genes (expressed in 14-16 tissues). The analysis was performed against a background list of 10,599 genes identified by David out of 17,214 genes expressed in 1-16 tissues.

GO Term	Count	FDR corrected p-value
GO:0044267 cellular protein metabolic process	1736	1.14E-52
GO:0016070 RNA metabolic process	764	5.68E-47
GO:0006412 translation	284	4.33E-38
GO:0006396 RNA processing	466	1.07E-37
GO:0008380 RNA splicing	254	6.10E-29
GO:0015031 protein transport	609	2.78E-27
GO:0016071 mRNA metabolic process	320	8.40E-27
GO:0006397 mRNA processing	281	9.56E-26
GO:0006886 intracellular protein transport	319	6.62E-21
GO:0043632 modification-dependent macromolecule catabolic process	452	6.82E-21
GO:0044257 cellular protein catabolic process	472	8.30E-21
GO:0030163 protein catabolic process	486	9.76E-21
GO:0034660 ncRNA metabolic process	193	5.31E-15
GO:0006464 protein modification process	1019	6.59E-13
GO:0045333 cellular respiration	94	7.66E-13
GO:0034470 ncRNA processing	158	8.43E-13
GO:0006605 protein targeting	184	4.71E-12
GO:0006414 translational elongation	88	7.31E-12
GO:0048193 Golgi vesicle transport	117	1.98E-11
GO:0016568 chromatin modification	220	1.06E-10
GO:0006457 protein folding	139	1.16E-08
GO:0022904 respiratory electron transport chain	62	1.75E-08
GO:0006351 transcription, DNA-dependent	220	2.28E-08
GO:0051169 nuclear transport	133	2.42E-08
GO:0032774 RNA biosynthetic process	222	2.71E-08
GO:0006913 nucleocytoplasmic transport	132	3.17E-08
GO:0010605 negative regulation of macromolecule metabolic process	515	3.60E-08
GO:0006281 DNA repair	215	3.64E-08
GO:0031324 negative regulation of cellular metabolic process	507	4.05E-08
GO:0051246 regulation of protein metabolic process	399	4.63E-08
GO:0022618 ribonucleoprotein complex assembly	64	4.90E-08
GO:0016310 phosphorylation	575	6.26E-08
GO:0050657 nucleic acid transport	84	6.74E-08
GO:0050658 RNA transport	84	6.74E-08
GO:0006259 DNA metabolic process	359	1.60E-07
GO:0033365 protein localization in organelle	120	3.22E-07
GO:0042773 ATP synthesis coupled electron transport	54	3.31E-07
GO:0051028 mRNA transport	75	3.46E-07
GO:0017038 protein import	110	3.53E-07

GO:0032268 regulation of cellular protein metabolic process	345	4.60E-07
GO:0032269 negative regulation of cellular protein metabolic process	145	5.99E-07
GO:0051248 negative regulation of protein metabolic process	148	2.20E-06
GO:0006352 transcription initiation	70	5.12E-06
