# Supplementary material for: Comparative Analysis of Human Tissue Interactomes Reveals Factors Leading to Tissue-Specific Manifestation of Hereditary Diseases
Source: PLoS Comput Biol. 2014 Jun 12;10(6):e1003632. doi: 10.1371/journal.pcbi.1003632 (PMC4055280; doi:10.1371/journal.pcbi.1003632)
Supplement: Table S3 — GO enrichment of tissue-specific genes relative to all expressed genes. (PDF) [file pcbi.1003632.s011.pdf]

Table S3: GO enrichment of tissue-specific genes relative to all expressed genes. Analysis was performed using the DAVID functional annotation chart with GO term Level of 5. Only terms with a corrected p-value < 10^{-5} with at least 50 genes in the query set are reported. DAVID identified 1,135 genes out of 2,411 tissue-specific genes (expressed in 1-3 tissues). The analysis was performed against a background list of 10,599 identified by David out of 17,214 genes expressed in 1-16 tissues.

**GO Term**	**Count**	**FDR corrected p-value**
GO:0042742 defense response to bacterium	34	2.02E-08
GO:0007283 spermatogenesis	63	7.30E-06
GO:0030001 metal ion transport	84	2.35E-05
GO:0007186 G-protein coupled receptor protein signaling pathway	98	5.82E-05
GO:0006812 cation transport	92	1.45E-04
GO:0007600 sensory perception	61	7.24E-04
GO:0006820 anion transport	34	6.69E-04
