# Supplementary material for: Comparative Analysis of Human Tissue Interactomes Reveals Factors Leading to Tissue-Specific Manifestation of Hereditary Diseases
Source: PLoS Comput Biol. 2014 Jun 12;10(6):e1003632. doi: 10.1371/journal.pcbi.1003632 (PMC4055280; doi:10.1371/journal.pcbi.1003632)
Supplement: Table S4 — GO enrichment of backbone proteins relative to all interactome proteins. (PDF) [file pcbi.1003632.s012.pdf]

Table S4: GO enrichment of core proteins relative to all interactome proteins. Analysis was performed using the DAVID functional annotation chart with GO term Level of 5. Only terms with a corrected p-value < 10^{-5} with at least 50 genes in the query set are reported. DAVID identified 3,899 genes out of the 4,989 genes in the core interactome. The analysis was performed against a background list of 7,909 genes identified by DAVID out of 11,225 genes in the global interactome.

GO Term	Count	FDR corrected p-value
GO:0016070 RNA metabolic process	584	2.77E-44
GO:0044267 cellular protein metabolic process	1167	1.95E-36
GO:0006396 RNA processing	361	7.63E-36
GO:0008380 RNA splicing	220	1.67E-30
GO:0006397 mRNA processing	239	1.51E-30
GO:0016071 mRNA metabolic process	268	1.43E-30
GO:0044257 cellular protein catabolic process	373	1.44E-22
GO:0030163 protein catabolic process	380	3.84E-22
GO:0043632 modification-dependent macromolecule catabolic process	356	4.36E-22
GO:0006412 translation	207	5.90E-22
GO:0015031 protein transport	438	4.32E-21
GO:0006886 intracellular protein transport	250	1.57E-16
GO:0016568 chromatin modification	182	2.57E-10
GO:0048193 Golgi vesicle transport	99	7.93E-10
GO:0006352 transcription initiation	67	2.46E-08
GO:0006414 translational elongation	74	2.43E-08
GO:0006605 protein targeting	141	1.03E-07
GO:0006351 transcription, DNA-dependent	180	1.06E-07
GO:0022618 ribonucleoprotein complex assembly	58	1.62E-07
GO:0032774 RNA biosynthetic process	181	1.91E-07
GO:0034660 ncRNA metabolic process	127	3.28E-07
GO:0051351 positive regulation of ligase activity	62	7.11E-07
GO:0006508 proteolysis	446	8.66E-07
GO:0051436 negative regulation of ubiquitin-protein ligase activity during mitotic cell cycle	56	1.29E-06
GO:0051437 positive regulation of ubiquitin-protein ligase activity during mitotic cell cycle	58	1.47E-06
GO:0034470 ncRNA processing	106	1.97E-06
GO:0051352 negative regulation of ligase activity	57	2.24E-06
GO:0031400 negative regulation of protein modification process	87	2.66E-06
GO:0051438 regulation of ubiquitin-protein ligase activity	64	4.75E-06
GO:0045333 cellular respiration	65	1.84E-05
GO:0032269 negative regulation of cellular protein metabolic process	117	2.37E-05
GO:0050657 nucleic acid transport	68	2.56E-05
GO:0050658 RNA transport	68	2.56E-05
GO:0006281 DNA repair	167	2.94E-05
GO:0010608 posttranscriptional regulation of gene expression	124	3.00E-05
GO:0033365 protein localization in organelle	93	4.97E-05
GO:0006464 protein modification process	647	5.59E-05

GO:0051246 regulation of protein metabolic process	295	7.59E-05
GO:0006457 protein folding	96	9.33E-05
