# Supplementary material for: Comparative Analysis of Human Tissue Interactomes Reveals Factors Leading to Tissue-Specific Manifestation of Hereditary Diseases
Source: PLoS Comput Biol. 2014 Jun 12;10(6):e1003632. doi: 10.1371/journal.pcbi.1003632 (PMC4055280; doi:10.1371/journal.pcbi.1003632)
Supplement: Table S5 — GO enrichment of tissue hubs versus the global interactome. (PDF) [file pcbi.1003632.s013.pdf]

Table S5: GO enrichment of tissue hubs versus the global interactome. Analysis was performed using the DAVID functional annotation chart with GO term Level of 5. Only terms with a corrected p-value < 10^{-5} with at least 50 genes in the query set are reported. DAVID identified 386 genes out of the 451 tissue hubs. The analysis was performed against a background list of 7,909 genes identified by DAVID out of 11,225 genes in the global interactome.

GO Term	Count	FDR corrected p-value
GO:0043067 regulation of programmed cell death	107	1.09E-24
GO:0042981 regulation of apoptosis	106	1.01E-24
GO:0006464 protein modification process	136	4.37E-23
GO:0051246 regulation of protein metabolic process	84	4.15E-23
GO:0010604 positive regulation of macromolecule metabolic process	104	4.87E-21
GO:0031325 positive regulation of cellular metabolic process	104	2.36E-20
GO:0010605 negative regulation of macromolecule metabolic process	91	6.82E-19
GO:0031324 negative regulation of cellular metabolic process	89	1.54E-18
GO:0044267 cellular protein metabolic process	170	3.76E-18
GO:0031326 regulation of cellular biosynthetic process	183	8.48E-18
GO:0043068 positive regulation of programmed cell death	66	9.56E-18
GO:0010556 regulation of macromolecule biosynthetic process	178	9.68E-18
GO:0010942 positive regulation of cell death	66	1.08E-17
GO:0043065 positive regulation of apoptosis	65	2.27E-17
GO:0010468 regulation of gene expression	178	3.36E-17
GO:0019219 regulation of nucleobase, nucleoside, nucleotide and nucleic acid metabolic process	172	7.07E-16
GO:0045449 regulation of transcription	162	7.31E-16
GO:0032268 regulation of cellular protein metabolic process	65	3.84E-15
GO:0009891 positive regulation of biosynthetic process	80	4.54E-15
GO:0031328 positive regulation of cellular biosynthetic process	79	9.44E-15
GO:0043066 negative regulation of apoptosis	55	8.99E-15
GO:0043069 negative regulation of programmed cell death	55	2.58E-14
GO:0051173 positive regulation of nitrogen compound metabolic process	76	2.45E-14
GO:0060548 negative regulation of cell death	55	2.35E-14
GO:0051252 regulation of RNA metabolic process	122	6.03E-14
GO:0010557 positive regulation of macromolecule biosynthetic process	75	1.01E-13
GO:0010629 negative regulation of gene expression	64	1.54E-13
GO:0009966 regulation of signal transduction	83	1.68E-13
GO:0051253 negative regulation of RNA metabolic process	52	4.16E-13
GO:0006355 regulation of transcription, DNA-dependent	117	7.42E-13
GO:0045941 positive regulation of transcription	67	1.04E-12
GO:0051172 negative regulation of nitrogen compound metabolic process	63	1.14E-12
GO:0010628 positive regulation of gene expression	68	1.23E-12

GO:0045935 positive regulation of nucleobase, nucleoside, nucleotide and nucleic acid metabolic process	71	1.45E-12
GO:0045934 negative regulation of nucleobase, nucleoside, nucleotide and nucleic acid metabolic process	62	2.20E-12
GO:0016481 negative regulation of transcription	58	2.37E-12
GO:0045892 negative regulation of transcription, DNA-dependent	50	3.05E-12
GO:0010558 negative regulation of macromolecule biosynthetic process	64	3.19E-12
GO:0009890 negative regulation of biosynthetic process	65	7.44E-12
GO:0031327 negative regulation of cellular biosynthetic process	64	9.33E-12
GO:0012502 induction of programmed cell death	46	3.06E-11
GO:0045893 positive regulation of transcription, DNA-dependent	57	8.66E-11
GO:0051254 positive regulation of RNA metabolic process	57	1.14E-10
GO:0030163 protein catabolic process	65	1.44E-10
GO:0044257 cellular protein catabolic process	64	1.42E-10
GO:0007242 intracellular signaling cascade	99	2.81E-10
GO:0043632 modification-dependent macromolecule catabolic process	59	4.08E-09
GO:0007243 protein kinase cascade	46	5.11E-09
GO:0006916 anti-apoptosis	33	8.67E-09
GO:0016310 phosphorylation	71	9.11E-09
GO:0051174 regulation of phosphorus metabolic process	53	9.95E-09
GO:0019220 regulation of phosphate metabolic process	53	9.95E-09
GO:0006259 DNA metabolic process	53	1.47E-08
