# Supplementary material for: Comparative Analysis of Human Tissue Interactomes Reveals Factors Leading to Tissue-Specific Manifestation of Hereditary Diseases
Source: PLoS Comput Biol. 2014 Jun 12;10(6):e1003632. doi: 10.1371/journal.pcbi.1003632 (PMC4055280; doi:10.1371/journal.pcbi.1003632)
Supplement: Table S6 — GO enrichment of global hubs versus global genes. (PDF) [file pcbi.1003632.s014.pdf]

Table S6: GO enrichment of global hubs versus global genes. Analysis was performed using the DAVID functional annotation chart with GO term Level of 5. Only terms with a corrected p-value < 10^{-5} with at least 50 genes in the query set are reported. DAVID identified 207 genes out of the 221 global hubs. The analysis was performed against a background list of 3,931 genes identified by DAVID out of 5,033 genes in all 16 tissues.

GO Term	Count	FDR corrected p-value
GO:0031328 positive regulation of cellular biosynthetic process	52	1.38E-13
GO:0009891 positive regulation of biosynthetic process	52	2.08E-13
GO:0042981 regulation of apoptosis	58	1.43E-12
GO:0031325 positive regulation of cellular metabolic process	60	1.56E-12
GO:0043067 regulation of programmed cell death	58	1.27E-12
GO:0051173 positive regulation of nitrogen compound metabolic process	50	1.36E-12
GO:0010604 positive regulation of macromolecule metabolic process	59	1.38E-12
GO:0051252 regulation of RNA metabolic process	73	2.00E-11
GO:0019219 regulation of nucleobase, nucleoside, nucleotide and nucleic acid metabolic process	101	2.38E-11
GO:0031326 regulation of cellular biosynthetic process	104	2.21E-11
GO:0010556 regulation of macromolecule biosynthetic process	102	1.10E-10
GO:0006355 regulation of transcription, DNA-dependent	69	1.89E-10
GO:0045449 regulation of transcription	94	1.93E-10
GO:0010468 regulation of gene expression	102	3.00E-10
GO:0006464 protein modification process	74	5.70E-10
GO:0010605 negative regulation of macromolecule metabolic process	51	7.60E-09
GO:0007242 intracellular signaling cascade	53	4.50E-06
GO:0044267 cellular protein metabolic process	96	8.37E-06
