# Supplementary material for: Comparative Analysis of Human Tissue Interactomes Reveals Factors Leading to Tissue-Specific Manifestation of Hereditary Diseases
Source: PLoS Comput Biol. 2014 Jun 12;10(6):e1003632. doi: 10.1371/journal.pcbi.1003632 (PMC4055280; doi:10.1371/journal.pcbi.1003632)
Supplement: Table S7 — Distribution of the number of hereditary diseases and their causal germline-aberrant disease genes by number of disease tissues they affect shows that most hereditary diseases are tissue-specific. (PDF) [file pcbi.1003632.s015.pdf]

Table S7: Distribution of the number of hereditary diseases and their causal germline-aberrant disease genes by number of disease tissues they affect shows that most hereditary diseases are tissue-specific.

Number of affected tissues	Number of hereditary diseases	Number of causal genes
1	293	182
2	6	39
3	3	10
4	1	1
