# Supplementary material for: Comparative Analysis of Human Tissue Interactomes Reveals Factors Leading to Tissue-Specific Manifestation of Hereditary Diseases
Source: PLoS Comput Biol. 2014 Jun 12;10(6):e1003632. doi: 10.1371/journal.pcbi.1003632 (PMC4055280; doi:10.1371/journal.pcbi.1003632)
Supplement: Table S8 — Tissue-selective hereditary diseases and their tissue-specific PPIs in their disease tissues. (PDF) [file pcbi.1003632.s016.pdf]

Table S8: Hereditary diseases, their causal genes and their tissue-specific PPIs in their disease tissues. An interaction is tissue-specific if it occurs in at most 3 tissues.

Disease Name	Causal gene symbol	Interactor gene symbol	Disease tissue with tissue-specific PPI	Other tissues with PPI	Number of causal gene PPIs in disease tissues
46XY partial gonadal dysgenesis with minifascicular neuropathy	DHH	HHIP	testis		1
Afibrinogenemia congenital	FGA	HRG	kidney, liver		17
Afibrinogenemia congenital	FGA	APOA1	kidney		17
Afibrinogenemia congenital	FGA	KLK6	kidney	brain	17
Afibrinogenemia congenital	FGB	LPA	liver	kidney	7
Alzheimer disease 1 familial	APP	SNCB	brain		86
Alzheimer disease 1 familial	APP	GFAP	brain	lung	86
Alzheimer disease 1 familial	APP	SHC3	brain		86
Alzheimer disease type 3 with spastic paraparesis and unusual plaques	PSEN1	ICAM5	brain	placenta, lung	58
Alzheimer disease type 3 with spastic paraparesis and unusual plaques	PSEN1	GFAP	brain	lung	58
Alzheimer disease-4	PSEN2	ICAM5	brain	placenta, lung	27
Alzheimer disease-4	PSEN2	GFAP	brain	lung	27
Amyloidosis hereditary transthyretin-related	TTR	APOA1	kidney		24
Amyloidosis renal	APOA1	CP	kidney		25
Amyloidosis renal	APOA1	TF	kidney		25
Amyloidosis renal	APOA1	APOL1	kidney		25
Amyloidosis renal	APOA1	PTPN5	kidney		25
Amyloidosis renal	APOA1	GPLD1	kidney		25
Amyloidosis renal	APOA1	PDE1A	kidney		25
Amyloidosis renal	APOA1	FN1	kidney		25
Amyloidosis renal	APOA1	TTR	kidney		25
Amyloidosis renal	APOA1	PCMT1	kidney		25
Amyloidosis renal	APOA1	CLU	kidney		25
Amyloidosis renal	APOA1	LBP	kidney		25
Amyloidosis renal	APOA1	LECT1	kidney		25
Amyloidosis renal	APOA1	APP	kidney		25
Amyloidosis renal	APOA1	SPEF2	kidney		25
Amyloidosis renal	APOA1	ROBO3	kidney		25
Amyloidosis renal	APOA1	C1QC	kidney		25
Amyloidosis renal	APOA1	RRP1B	kidney		25
Amyloidosis renal	APOA1	ALB	kidney		25
Amyloidosis renal	APOA1	KRT9	kidney		25
Amyloidosis renal	APOA1	FGA	kidney		25
Amyloidosis renal	APOA1	SMPDL3A	kidney		25

Amyloidosis renal	APOA1	TNFRSF10C	kidney		25
Amyloidosis renal	APOA1	TOMM20	kidney		25
Amyloidosis renal	APOA1	PDE4B	kidney		25
Amyloidosis renal	FGA	HRG	kidney,liver		17
Amyloidosis renal	FGA	APOA1	kidney		17
Amyloidosis renal	FGA	KLK6	kidney	brain	17
Androgen insensitivity	AR	POU2F2	testis		160
Androgen insensitivity	AR	MAGEA11	testis		160
Androgen insensitivity	AR	DCC	testis		160
Antley-Bixler syndrome without genital anomalies or disordered steroidogenesis	FGFR2	FGF9	brain,ovary,kidney	prostate	8
Antley-Bixler syndrome without genital anomalies or disordered steroidogenesis	FGFR2	FGF1	brain, kidney	colon, breast	7
Apert syndrome	FGFR2	FGF9	brain,ovary,kidney	prostate	8
Apert syndrome	FGFR2	FGF1	brain, kidney	colon, breast	7
Atrial septal defect 2	GATA4	NKX2-5	heart	thyroid	13
Atrial septal defect 7 with or without AV conduction defects	NKX2-5	TBX5	heart	thyroid	7
Atrial septal defect 7 with or without AV conduction defects	NKX2-5	CAMTA2	heart	thyroid	7
Atrial septal defect 7 with or without AV conduction defects	NKX2-5	ID2	heart	thyroid	7
Atrial septal defect 7 with or without AV conduction defects	NKX2-5	HDAC1	heart	thyroid	7
Atrial septal defect 7 with or without AV conduction defects	NKX2-5	ID3	heart	thyroid	7
Atrial septal defect 7 with or without AV conduction defects	NKX2-5	ID1	heart	thyroid	7
Atrial septal defect 7 with or without AV conduction defects	NKX2-5	GATA4	heart	thyroid	7
Becker muscular dystrophy	DMD	SNTA1	Skeletal muscle		11
Becker muscular dystrophy	DMD	DTNA	Skeletal muscle l		11
Becker muscular dystrophy	DMD	ARRB1	Skeletal muscle		11
Becker muscular dystrophy	DMD	ACTA1	Skeletal muscle, heart		11
Becker muscular dystrophy	DMD	ANK2	Skeletal muscle		11
Becker muscular dystrophy	DMD	ALB	Skeletal muscle, heart		11
Becker muscular dystrophy	DMD	SNTB2	Skeletal muscle		11
Becker muscular dystrophy	DMD	SNTB1	Skeletal muscle, heart		11
Becker muscular dystrophy	DMD	DAG1	Skeletal muscle, heart		11

Becker muscular dystrophy	DMD	KCNJ12	Skeletal muscle, heart		11
Becker muscular dystrophy	DMD	MAP3K5	Skeletal muscle		11
Campomelic dysplasia	SOX9	NR5A1	Testis	ovary	17
Cardiomyopathy dilated 1A	MYBPC3	SMURF2	Heart		4
Cardiomyopathy dilated 1A	MYBPC3	TTN	Heart		4
Cardiomyopathy dilated 1A	MYBPC3	TRIM63	Heart		4
Cardiomyopathy dilated 1A	MYBPC3	ALK	Heart		4
Cardiomyopathy dilated 1G	TTN	MYBPC3	heart		14
Cardiomyopathy dilated 3B	DMD	SNTA1	Skeletal muscle		11
Cardiomyopathy dilated 3B	DMD	DTNA	Skeletal muscle		11
Cardiomyopathy dilated 3B	DMD	ARRB1	Skeletal muscle		11
Cardiomyopathy dilated 3B	DMD	ACTA1	Skeletal muscle, heart		11
Cardiomyopathy dilated 3B	DMD	ANK2	Skeletal muscle		11
Cardiomyopathy dilated 3B	DMD	ALB	Skeletal muscle, heart		11
Cardiomyopathy dilated 3B	DMD	SNTB2	Skeletal muscle		11
Cardiomyopathy dilated 3B	DMD	SNTB1	Skeletal muscle, heart		11
Cardiomyopathy dilated 3B	DMD	DAG1	Skeletal muscle, heart		11
Cardiomyopathy dilated 3B	DMD	KCNJ12	Skeletal muscle, heart		11
Cardiomyopathy dilated 3B	DMD	MAP3K5	Skeletal muscle		11
Cardiomyopathy familial restrictive	TNNI3	SMURF2	Heart	testis, skeletal muscle	7
Cardiomyopathy familial restrictive	TNNI3	TNNC1	Heart	testis, skeletal muscle	7
Cardiomyopathy familial restrictive	TNNI3	TNNT2	Heart	testis, skeletal muscle	7
Cardiomyopathy familial restrictive	TNNI3	PKD2	Heart	testis, skeletal muscle	7
Cardiomyopathy familial restrictive	TNNI3	TRIM21	Heart	testis, skeletal muscle	7
Cardiomyopathy familial restrictive	TNNI3	LYST	Heart	testis	7
Cardiomyopathy familial restrictive	TNNI3	TRIM63	heart	testis, skeletal muscle	7

Cardiomyopathy hypertrophic midventricular digenic	CAV3	DYSF	Skeletal muscle, heart	kidney, testis	8
Cardiomyopathy hypertrophic midventricular digenic	CAV3	JPH2	Skeletal muscle		8
Chorea hereditary benign	NKX2-1	CREBBP	Thyroid	lung, liver	17
Chorea hereditary benign	NKX2-1	PAX8	thyroid	Lung	17
Chorea hereditary benign	NKX2-1	RARA	thyroid	lung, liver	17
Chorea hereditary benign	NKX2-1	NCOA2	thyroid	lung, liver	17
Chorea hereditary benign	NKX2-1	CRK	thyroid	appendix, liver	17
Chorea hereditary benign	NKX2-1	PTRF	thyroid	lung	17
Choroid plexus papilloma	TP53	RASGRF1	brain		291
Choroid plexus papilloma	TP53	CITED1	breast, brain	thyroid, testis	306
Congenital bilateral absence of vas deferens	CFTR	SLC4A8	lung	brain, ovary	138
Congenital bilateral absence of vas deferens	CFTR	SH3BGRL2	lung	colon, adrenal	138
Congenital bilateral absence of vas deferens	CFTR	HSPA1L	testis		115
Congenital bilateral absence of vas deferens	CFTR	HSPA1L	testis		115
Craniofacial-deafness-hand syndrome	PAX3	MEOX1	Skeletal muscle	adrenal, thyroid	14
Creatine phosphokinase elevated serum	CAV3	DYSF	Skeletal muscle, heart	kidney, testis	8
Creatine phosphokinase elevated serum	CAV3	JPH2	Skeletal muscle		8
Creutzfeldt-Jakob disease	PRNP	C18orf56	Brain	testis	50
Cystic fibrosis	CFTR	SLC4A8	lung	brain, ovary	138
Cystic fibrosis	CFTR	SH3BGRL2	lung	colon, adrenal	138
Cystic fibrosis	CFTR	HSPA1L	testis		115
Cystic fibrosis	CFTR	HSPA1L	testis		115
Dementia frontotemporal with or without parkinsonism	PSEN1	ICAM5	brain	placenta, lung	58
Dementia frontotemporal with or without parkinsonism	PSEN1	GFAP	brain	lung	58
Dementia frontotemporal with or without parkinsonism	MAPT	SLC1A2	brain	liver	31
Dementia Lewy body	SNCA	SNCB	brain		58
Dementia Lewy body	SNCA	PAK3	brain	thyroid, testis	58
Duchenne muscular dystrophy	DMD	SNTA1	Skeletal muscle		11
Duchenne muscular dystrophy	DMD	DTNA	Skeletal muscle		11
Duchenne muscular dystrophy	DMD	ARRB1	Skeletal muscle		11
Duchenne muscular dystrophy	DMD	ACTA1	Skeletal muscle, heart		11

Duchenne muscular dystrophy	DMD	ANK2	Skeletal muscle		11
Duchenne muscular dystrophy	DMD	ALB	Skeletal muscle, heart		11
Duchenne muscular dystrophy	DMD	SNTB2	Skeletal muscle		11
Duchenne muscular dystrophy	DMD	SNTB1	Skeletal muscle, heart		11
Duchenne muscular dystrophy	DMD	DAG1	Skeletal muscle, heart		11
Duchenne muscular dystrophy	DMD	KCNJ12	Skeletal muscle, heart		11
Duchenne muscular dystrophy	DMD	MAP3K5	Skeletal muscle		11
Dystonia DOPA-responsive with or without hyperphenylalainemia	GCH1	RPH3A	brain, liver	wbc, lung	9
Dystonia DOPA-responsive with or without hyperphenylalainemia	GCH1	RAB26	brain		9
Epilepsy nocturnal frontal lobe 1	CHRNA4	YWHAH	brain		1
Episodic ataxia type 2	CACNA1A	SYT1	brain	heart, adrenal	5
Episodic ataxia type 2	CACNA1A	CABP1	brain	heart	5
Episodic ataxia type 2	CACNA1A	CACNB4	brain	heart, adrenal	5
Gerstmann-Straussler disease	PRNP	C18orf56	brain	testis	50
Hemophagocytic lymphohistiocytosis familial 2	PRF1	DDX24	wbc	Bone marrow, lung	4
Hemophagocytic lymphohistiocytosis familial 2	PRF1	GZMB	wbc	Bone marrow, lung	4
Hemophagocytic lymphohistiocytosis familial 2	PRF1	SRGN	wbc	Bone marrow, lung	4
Hemophagocytic lymphohistiocytosis familial 2	PRF1	CALR	wbc	Bone marrow, lung	4
Huntington disease-like 1	PRNP	C18orf56	brain	testis	50
Hypercholesterolemia due to ligand-defective apo B	APOB	MTTP	liver	Small intestine	11
Hypercholesterolemia due to ligand-defective apo B	APOB	LIPC	liver	kidney	11
Hypercholesterolemia familial	LDLR	PCSK9	liver	lung	10
Hypercholesterolemia familial 3	PCSK9	LDLR	liver	lung	1
Hyperferritinemia-cataract syndrome	FTL	KNG1	liver	kidney, testis	14
Hyperpehnylalaninemia BH4-deficient B	GCH1	RPH3A	brain,liver	wbc, lung	9
Hyperpehnylalaninemia BH4-deficient B	GCH1	RAB26	brain		9

Hypoalphalipoproteinemia	APOA1	CP	kidney		25
Hypoalphalipoproteinemia	APOA1	TF	kidney		25
Hypoalphalipoproteinemia	APOA1	APOL1	kidney		25
Hypoalphalipoproteinemia	APOA1	PTPN5	kidney		25
Hypoalphalipoproteinemia	APOA1	GPLD1	kidney		25
Hypoalphalipoproteinemia	APOA1	PDE1A	kidney		25
Hypoalphalipoproteinemia	APOA1	FN1	kidney		25
Hypoalphalipoproteinemia	APOA1	TTR	kidney		25
Hypoalphalipoproteinemia	APOA1	PCMT1	kidney		25
Hypoalphalipoproteinemia	APOA1	CLU	kidney		25
Hypoalphalipoproteinemia	APOA1	LBP	kidney		25
Hypoalphalipoproteinemia	APOA1	LECT1	kidney		25
Hypoalphalipoproteinemia	APOA1	APP	kidney		25
Hypoalphalipoproteinemia	APOA1	SPEF2	kidney		25
Hypoalphalipoproteinemia	APOA1	ROBO3	kidney		25
Hypoalphalipoproteinemia	APOA1	C1QC	kidney		25
Hypoalphalipoproteinemia	APOA1	RRP1B	kidney		25
Hypoalphalipoproteinemia	APOA1	ALB	kidney		25
Hypoalphalipoproteinemia	APOA1	KRT9	kidney		25
Hypoalphalipoproteinemia	APOA1	FGA	kidney		25
Hypoalphalipoproteinemia	APOA1	SMPDL3A	kidney		25
Hypoalphalipoproteinemia	APOA1	TNFRSF1 0C	kidney		25
Hypoalphalipoproteinemia	APOA1	TOMM20	kidney		25
Hypoalphalipoproteinemia	APOA1	PDE4B	kidney		25
Hypoalphalipoproteinemia	APOA1	not found	kidney		25
Hypochondroplasia	FGFR3	FGF9	brain		22
Hypochondroplasia	FGFR3	FGF1	brain	colon	22
Hypochondroplasia	FGFR3	CTSK	brain	skin, adrenal	22
Hypogonadotropic hypogonadism	FGFR1	FGF1	brain	heart, adipose	18
Hypokalemic periodic paralysis type 1	CACNA1 S	SRI	skeletal,t hyroid	breast	1
Insomnia fatal familial	PRNP	C18orf56	brain	testis	50
Jackson-Weiss syndrome	FGFR2	FGF9	brain, ovary, kidney	prostate	8
Jackson-Weiss syndrome	FGFR2	FGF1	brain, kidney	colon, breast	7
Jackson-Weiss syndrome	FGFR1	FGF1	brain	heart, adipose	18
Leukoencephalopathy with vanishing white matter	EIF2B1	DCC	brain	testis	10
Leukoencephalopathy with vanishing white matter	EIF2B4	DCC	brain	testis	4
Leukoencephalopathy with vanishing white matter	EIF2B2	DCC	brain	testis	5
Li-Fraumeni syndrome	CHEK2	GINS2	adrenal	testis	26
Li-Fraumeni-like syndrome	TP53	RASGRF1	brain		291
Li-Fraumeni-like syndrome	TP53	CITED1	breast, brain	thyroid, testis	306
Migraine familial hemiplegic 1 with progressive cerebellar ataxia	CACNA1 A	SYT1	brain	heart, adrenal	5

Migraine familial hemiplegic 1 with progressive cerebellar ataxia	CACNA1A	CABP1	brain	heart	5
Migraine familial hemiplegic 1 with progressive cerebellar ataxia	CACNA1A	CACNB4	brain	heart, adrenal	5
Mismatch repair cancer syndrome	MLH1	PTPRH	colon	liver	132
Mismatch repair cancer syndrome	MSH2	ESR2	ovary	testis, breast	31
Mismatch repair cancer syndrome	PMS2	PSD2	brain		37
Muscular dystrophy limb-girdle type 2J	TTN	MYBPC3	heart		14
Muscular dystrophy limb-girdle type IC	CAV3	DYSF	Skeletal muscle, heart	kidney, testis	8
Muscular dystrophy limb-girdle type IC	CAV3	JPH2	Skeletal muscle		8
Myasthenic syndrome congenital associated with facial dysmorphism and acetylcholine receptor deficiency	RAPSN	KHDRBS1	Skeletal muscle	thyroid	2
Myasthenic syndrome congenital associated with facial dysmorphism and acetylcholine receptor deficiency	RAPSN	UBE2Z	Skeletal muscle	thyroid	2
Myasthenic syndrome slow-channel congenital	CHRND	CHRNA1	Skeletal muscle	thyroid	1
Myasthenic syndrome slow-channel congenital	CHRNA1	CHRND	Skeletal muscle	thyroid	3
Myasthenic syndrome slow-channel congenital	CHRNA1	CHRNG	Skeletal muscle		3
Myopathy nemaline 3	ACTA1	DMD	Skeletal muscle	heart	38
Myopathy proximal with early respiratory muscle involvement	TTN	MYBPC3	heart		14
Neurodegeneration with brain iron accumulation 3	FTL	KNG1	liver	kidney, testis	14
Neuropathy distal hereditary motor type IIA	HSPB8	HSPB7	Skeletal muscle	heart, adipose	7
Oculodentodigital dysplasia	GJA1	GJA3	heart	kidney, testis	10
Ovarian dysgenesis 1	FSHR	GRK4	testis		5
Ovarian dysgenesis 1	FSHR	YWHAQ	testis		5
Ovarian dysgenesis 1	FSHR	UBC	testis		5
Ovarian dysgenesis 1	FSHR	ADRBK1	testis		5
Ovarian dysgenesis 1	FSHR	GRK6	testis		5
Ovarian hyperstimulation syndrome	FSHR	GRK4	testis		5
Ovarian hyperstimulation syndrome	FSHR	YWHAQ	testis		5
Ovarian hyperstimulation syndrome	FSHR	UBC	testis		5
Ovarian hyperstimulation	FSHR	ADRBK1	testis		5

syndrome					
Ovarian hyperstimulation syndrome	FSHR	GRK6	testis		5
Ovarioleukodystrophy	EIF2B4	DCC	brain	testis	4
Parkes Weber slndrome	RASA1	SYN1	brain		36
Parkinson disease 1,4	SNCA	SNCB	brain		58
Parkinson disease 1,4	SNCA	SNCB	brain		58
Parkinson disease 1,4	SNCA	PAK3	brain	thyroid, testis	58
Parkinson disease 1,4	SNCA	PAK3	brain	thyroid, testis	58
Pick disease	PSEN1	ICAM5	brain	placenta, lung	58
Pick disease	PSEN1	GFAP	brain	lung	58
Pick disease	MAPT	SLC1A2	brain	liver	31
Prion disease with protracted course	PRNP	C18orf56	brain	testis	50
Pyruvate kinase deficiency	PKLR	USPL1	liver	brain, kidney	6
Pyruvate kinase deficiency	PKLR	USP3	liver	brain, kidney	6
Pyruvate kinase deficiency	PKLR	CLEC4G	liver		6
Rippling muscle disease	CAV3	DYSF	Skeletal muscle, heart	kidney, testis	8
Rippling muscle disease	CAV3	JPH2	Skeletal muscle		8
Severe combined immunodeficiency T-cell negative B-cell/natural killer cell-positive type	PTPRC	GHR	wbc	Lymph node, lung	29
Severe combined immunodeficiency T-cell negative B-cell/natural killer cell-positive type	PTPRC	CD1D	wbc	Lymph node	29
Severe combined immunodeficiency T-cell negative B-cell/natural killer cell-positive type	PTPRC	INSR	wbc	Lymph node, lung	29
Severe combined immunodeficiency T-cell negative B-cell/natural killer cell-positive type	PTPRC	DPP4	wbc	Lymph node, lung	29
Severe combined immunodeficiency T-cell negative B-cell/natural killer cell-positive type	PTPRC	PTPRCAP	wbc	Lymph node	29
Severe combined immunodeficiency X-linked	IL2RG	IL4R	wbc		7
Spinocerebellar ataxia 17	TBP	EMID2	brain		101
Spinocerebellar ataxia 17	TBP	POU3F2	brain		101
Spinocerebellar ataxia 6	CACNA1A	SYT1	brain	heart, adrenal	5
Spinocerebellar ataxia 6	CACNA1A	CABP1	brain	heart	5
Spinocerebellar ataxia 6	CACNA1A	CACNB4	brain	heart, adrenal	5
Supranuclear palsy	MAPT	SLC1A2	brain	liver	31

progressive					
Supranuclear palsy progressive	MAPT	SLC1A2	brain	liver	31
Supravalvar aortic stenosis	ELN	FBN2	heart	testis, adipose	9
Syndactyly type III	GJA1	GJA3	heart	kidney, testis	10
Tetrology of Fallot	NKX2-5	TBX5	heart	thyroid	7
Tetrology of Fallot	NKX2-5	CAMTA2	heart	thyroid	7
Tetrology of Fallot	NKX2-5	ID2	heart	thyroid	7
Tetrology of Fallot	NKX2-5	HDAC1	heart	thyroid	7
Tetrology of Fallot	NKX2-5	ID3	heart	thyroid	7
Tetrology of Fallot	NKX2-5	ID1	heart	thyroid	7
Tetrology of Fallot	NKX2-5	GATA4	heart	thyroid	7
Thanatophoric dysplasia type I,II	FGFR3	FGF9	brain		22
Thanatophoric dysplasia type I,II	FGFR3	FGF9	brain		22
Thanatophoric dysplasia type I,II	FGFR3	FGF1	brain	colon	22
Thanatophoric dysplasia type I,II	FGFR3	FGF1	brain	colon	22
Thanatophoric dysplasia type I,II	FGFR3	CTSK	brain	skin, adrenal	22
Thanatophoric dysplasia type I,II	FGFR3	CTSK	brain	skin, adrenal	22
Warfarin sensitivity	CYP2C9	CDC37	liver	colon, small intestine	2
Warfarin sensitivity	CYP2C9	POR	liver	colon, small intestine	2
Adenomatous polyposis coli; Turcot syndrome	APC	DLGAP1	brain	testis	193
Adenomatous polyposis coli; Turcot syndrome	APC	PAK3	brain	thyroid, testis	193
Ataxia-telangiectasia	ATM	TERF1	wbc	brain	61
Bloom Syndrome	BLM	TERF1	wbc	brain	32
Hereditary breast/ovarian cancer	BRCA1	ESR1	breast		152
Hereditary breast/ovarian cancer	BRCA1	HIST1H4 E	ovary	brain, prostate	158
familial breast cancer	CHEK2	GINS2	adrenal	testis	26
Familial lung cancer	EGFR	ROS1	lung	prostate, kidney	291
Familial lung cancer	EGFR	ZAP70	lung	Lymph node, adrenal	291
Familial lung cancer	EGFR	EREG	lung		291
Familial lung cancer	EGFR	KRT1	lung	adipose	291
Familial lung cancer	EGFR	KRT6A	lung	thyroid, adipose	291
Hereditary non-polyposis colorectal cancer, Turcot syndrome	MLH1	PTPRH	colon	liver	132
Hereditary non-polyposis colorectal cancer, Turcot	MLH1	MUC2	colon	thyroid	132

syndrome					
Hereditary non-polyposis colorectal cancer	MSH2	ESR2	ovary	testis, breast	31
Hereditary non-polyposis colorectal cancer, Turcot syndrome	PMS2	PSD2	brain		37
Familial retinoblastoma	RB1	GATA1	lung	testis, prostate	127
Li-Fraumeni syndrome	TP53	PIN1	adrenal, brain	testis, prostate	328
Li-Fraumeni syndrome	TP53	CITED1	breast, brain	thyroid, testis	306
Li-Fraumeni syndrome	TP53	RASGRF1	brain		291
Muscular Dystrophy	DAG1	CAV3	skeletal	heart, testis	14
