# Supplementary material for: Comparative Analysis of Human Tissue Interactomes Reveals Factors Leading to Tissue-Specific Manifestation of Hereditary Diseases
Source: PLoS Comput Biol. 2014 Jun 12;10(6):e1003632. doi: 10.1371/journal.pcbi.1003632 (PMC4055280; doi:10.1371/journal.pcbi.1003632)
Supplement: Table S9 — Overview of the numbers of genes and tissues measured in each dataset. (PDF) [file pcbi.1003632.s017.pdf]

Table S9: Overview of the numbers of genes and tissues measured in each dataset.

	GNF (transcript)	HPA (protein)	RNA-seq (transcript)
Measured objects	26,560 probe-sets	13,150 antibodies	N/A
ENSEMBL genes mapped to measured objects	16,052	10,100	24,433
Genes expressed (threshold)	10,651 (≥100)	8,967 (see Methods)	18,399 (≥1)
Protein-coding genes expressed	9,279	8,967	14,909
Profiled tissues	78	66	16
