# Supplementary material for: Comparative Analysis of Human Tissue Interactomes Reveals Factors Leading to Tissue-Specific Manifestation of Hereditary Diseases
Source: PLoS Comput Biol. 2014 Jun 12;10(6):e1003632. doi: 10.1371/journal.pcbi.1003632 (PMC4055280; doi:10.1371/journal.pcbi.1003632)
Supplement: Table S10 — Consolidation of tissues from the different datasets into 16 main tissues. (PDF) [file pcbi.1003632.s018.pdf]

Table S10: Consolidation of tissues from the different datasets into 16 main tissues. Brackets group together samples from similar or closely-related sub-tissues. The name of the dataset appears after the '@' sign.

Tissue Group	Tissue samples from different data sources
**Adipose**	(Adipocyte@GNF;adipose@bodymap)
**Adrenal**	AdrenalCortex@GNF<br>(Adrenalgland@GNF; Adrenal gland- glandular cells@HPA; adrenal@bodymap)
**Brain**	(Wholebrain@GNF; brain@bodymap)<br>(Cerebellum - Cells in granular layer@HPA; Cerebellum - Cells in molecular layer@HPA; Cerebellum - Purkinje cells@HPA; Cerebellum@GNF)<br>(PrefrontalCortex@GNF;TemporalLobe@GNF; Cerebral cortex - Glial cells@HPA; Cerebral cortex - Neuronal cells@HPA; Lateral ventricle - Glial cells@HPA; Lateral ventricle - Neuronal cells@HPA; PrefrontalCortex@GNF)<br>(Amygdala@GNF; CiliaryGanglion@GNF; CingulateCortex@GNF; Hypothalamus@GNF; Hippocampus - Glial cells@HPA; Hippocampus - Neuronal cells@HPA; Caudatenucleus@GNF)
**Breast**	(breast@bodymap, Breast - Glandular cells@HPA)
**Colon**	(colon@GNF; colon@bodymap); Colon - Glandular cells@HPA
**Heart**	(CardiacMyocytes@GNF; Heart muscle - Myocytes@HPA); (Heart@GNF; heart@bodymap); AtrioventricularNode@GNF
**Kidney**	(Kidney@GNF; Kidney@bodymap); Kidney - Cells in glomeruli@HPA; Kidney - Cells in tubules@HPA
**Liver**	Liver - Bile duct cells@HPA; (Liver@GNF; Liver@bodymap);Liver - Hepatocytes@HPA
**Lung**	(Lung@GNF;lung@bodymap); Lung - Pneumocytes@HPA; Lung - Macrophages@HPA
**Lymph Node**	Lymph node - Germinal center cells@HPA; (Lymphnode@GNF; LymphNode@bodymap); Lymph node - Non-germinal center cells@HPA
**Ovary**	(Ovary@GNF; ovary@bodymap); Ovary - Ovarian stroma cells@HPA; Ovary - Follicle cells@HPA
**Prostate**	(Prostate@GNF; Prostate@bodymap); Prostate - Glandular cells@HPA
**Skeletal muscle**	(SkeletalMuscle@GNF; Skeletal muscle - Myocytes@HPA; Muscle@bodymap)
**Testis**	(Testis@GNF;testes@bodymap); TestisGermCell@GNF; TestisIntersitial@GNF; (TestisSeminiferousTubule@GNF; Testis - Cells in seminiferus ducts@HPA); (TestisLeydigCell@GNF; Testis - Leydig cells@HPA)
**Thyroid**	(Thyroid@GNF; Thyroid@bodymap); Thyroid gland - Glandular cells@HPA
**White Blood Cells (WBC)**	(721_B_lymphoblasts@GNF; CD19+_BCells(neg._sel.)@GNF; CD56+_NKCells@GNF; CD4+_Tcells@GNF; CD8+_Tcells@GNF; (BDCA4+_DentriticCells@GNF; CD14+_Monocytes@GNF;CD33+_Myeloid@GNF); Wbc@bodymap;
