# Supplementary material for: Comparative Analysis of Human Tissue Interactomes Reveals Factors Leading to Tissue-Specific Manifestation of Hereditary Diseases
Source: PLoS Comput Biol. 2014 Jun 12;10(6):e1003632. doi: 10.1371/journal.pcbi.1003632 (PMC4055280; doi:10.1371/journal.pcbi.1003632)
Supplement: Table S11 — The distribution of the number of PPIs across 1–16 tissues. (PDF) [file pcbi.1003632.s019.pdf]

Table S11: The distribution of the number of PPIs across 1-16 tissues.

Number of tissues	Number of PPIs
1	3,357
2	1,931
3	2,015
4	1,666
5	1,333
6	1,215
7	1,126
8	1,043
9	1,241
10	1,407
11	1,668
12	1,823
13	2,629
14	4,234
15	6,582
16	26,370
