# Supplementary material for: Comparative Analysis of Human Tissue Interactomes Reveals Factors Leading to Tissue-Specific Manifestation of Hereditary Diseases
Source: PLoS Comput Biol. 2014 Jun 12;10(6):e1003632. doi: 10.1371/journal.pcbi.1003632 (PMC4055280; doi:10.1371/journal.pcbi.1003632)
Supplement: Table S12 — The numbers of genes and PPIs in the interactome of each tissue. (PDF) [file pcbi.1003632.s020.pdf]

Table S12: The numbers of genes and PPIs in the interactome of each tissue.

Tissue	Number of genes	Number of PPIs
Adipose	7,129	40,874
Adrenal	8,555	49,973
Brain	8,611	48,788
Breast	7,860	42,891
Colon	8,243	47,057
Heart	7,889	44,162
Kidney	8,342	46,926
Liver	7,299	39,651
Lung	8,539	48,653
Lymph Node	8,227	47,330
Ovary	7,859	43,718
Prostate	8,389	48,292
Skeletal Muscle	7,480	42,759
Testis	8,898	50,902
Thyroid	8,325	47,614
White Blood Cells	7,064	40,069
