# Supplementary material for: Comparative Analysis of Human Tissue Interactomes Reveals Factors Leading to Tissue-Specific Manifestation of Hereditary Diseases
Source: PLoS Comput Biol. 2014 Jun 12;10(6):e1003632. doi: 10.1371/journal.pcbi.1003632 (PMC4055280; doi:10.1371/journal.pcbi.1003632)
Supplement: Table S13 — The distribution of the number of expressed causal genes across 1–16 tissues. (PDF) [file pcbi.1003632.s021.pdf]

Table S13: The distribution of the number of causal genes expressed in 1-16 tissues.

Number of tissues	Number of expressed casual genes
1	8
2	9
3	6
4	7
5	9
6	4
7	6
8	1
9	9
10	6
11	5
12	16
13	15
14	23
15	24
16	85
