# Supplementary material for: Comparative Analysis of Human Tissue Interactomes Reveals Factors Leading to Tissue-Specific Manifestation of Hereditary Diseases
Source: PLoS Comput Biol. 2014 Jun 12;10(6):e1003632. doi: 10.1371/journal.pcbi.1003632 (PMC4055280; doi:10.1371/journal.pcbi.1003632)

Figure S2: The 16 tissue interactomes show similar distributions of PPI degrees. The PPI degree of a protein in a tissue is the number of its PPI partners in that tissue. Most genes have at most 5 PPI partners in a tissue. Also shown are the PPI degree distributions within the global interactome and within the backbone (core) interactome, which is shared by all tissues.

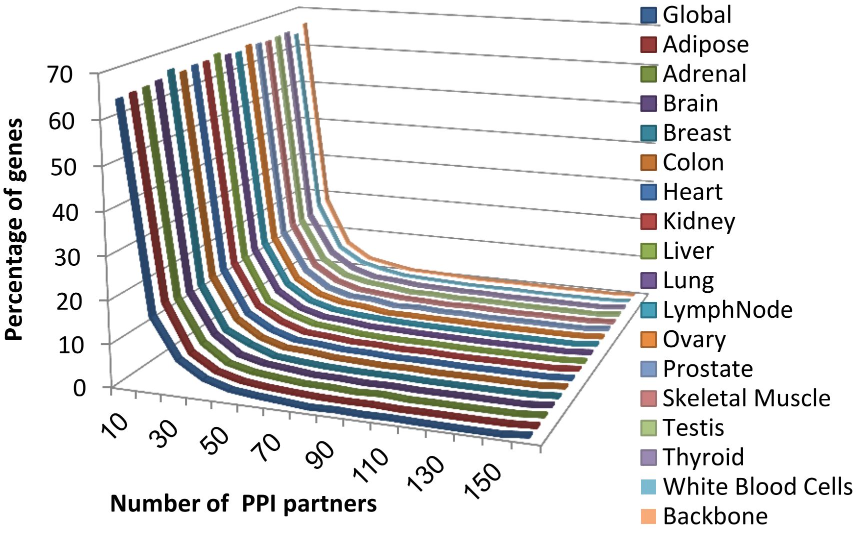

Supplement: Figure S2 — The 16 tissue interactomes show similar distributions of PPI degrees. The PPI degree of a protein in a tissue is the number of its PPI partners in that tissue. Most genes have at most 5 PPI partners in a tissue. Also shown are the PPI degree distributions within the global interactome and within the backbone interactome shared by all tissues. (PDF) [file pcbi.1003632.s002.pdf]
